# Supplementary material for: Unraveling the B. pseudomallei Heptokinase WcbL: From Structure to Drug Discovery
Source: Chem Biol. 2015 Dec 17;22(12):1622–32. doi: 10.1016/j.chembiol.2015.10.015 (PMC4691232; doi:10.1016/j.chembiol.2015.10.015)
Supplement: Document S1. Figures S1–S6 and Table S1 [file mmc1.pdf]

Chemistry & Biology, Volume *22*

Supplemental Information

Unraveling the *B. pseudomallei* Heptokinase

WcbL: From Structure to Drug Discovery

Mirella Vivoli, Michail N. Isupov, Rebecca Nicholas, Andrew Hill, Andrew E. Scott, Paul Kosma, Joann L. Prior, and Nicholas J. Harmer

Supplementary Table 1. Data collection and refinement statistics (Figure 2)

[a] Values in parentheses are given for the outer resolution shell.

[*] $R_{sym}=\Sigma_h\Sigma_J|<I_h>-I_J(h)|/\Sigma_h\Sigma_J I(h)$, where I(h) is the intensity of reflection h. Σ_h is the sum over all reflections and Σ_J is the sum over J measurements of the reflection.

[†] Rcryst =Σ||Fo|-|Fc||/Σ|Fo|

[‡] Target values are given in square brackets.

[§] Wilson B-factor was estimated by SFCHECK (Vaguine, et al., 1999).

[¶]Ramachandran plot analysis was performed by *MolProbity* (Chen, et al., 2010).

[#] Cryo5 consist of 12.5 % di-ethylene glycol + 25 % ethylene glycol + 12.5 % MPD + 12.5 % 1,2-propanediol + 12.5 % glycerol + 12.5 mM NDSB 201 (Molecular Dimensions).

**Dataset**	**Se-Met high energy remote**	**Glycerol complex**	**Native**	**Mannose complex**	**AMP-PNP complex**
Beamline (Diamond)	I03	I03	I04-1	I03	I03
Spacegroup	*C222₁*	*C222₁*	*C222₁*	*C222₁*	*C222₁*
Cell parameters (Å)	*a*=101.6, *b*=116.2, *c*=167.7	*a*=101.1, *b*=115.9, *c*=167.4	*a*=101.6, *b*=116.1, *c*=168.2	*a*=101.1 *b*=116.3, *c*=168.1	*a*=101.4 *b*=115.0, *c*=166.8
Protein sequence modification	Se-Met	-	E179A, E180A, E181A	E179A, E180A, E181A	E179A, E180A, E181A
Rotation interval collected °.	360	180	200	180	180
Wavelength (Å)	0.9778	0.9763	0.9200	0.9763	0.9763
Resolution range (Å)	56.5-2.27 (2.33-2.27)	76.2-1.82 (1.87-1.82)	48.6-1.76 (1.81-1.76)	69.5-1.94 (1.99-1.94)	76.1- 1.93 (1.98-1.93)
Completeness (%)	99.7 (99.7)	99.1 (93.8)[a]	98.4 (96.6)	99.6 (99.1)	99.6 (96.7)
Redundancy	14.6 (14.2)	7.2 (5.4)[a]	7.4 (7.6)	6.5 (6.4)	6.5 (6.3)
(I)/σ (I)	15.8 (3.7)	15.4 (1.9)[a]	12.6 (1.9) [a]	7.5 (2.2)	12.8 (2.1)
[*] R_{sym} (%)	0.127 (0.779)	0.078 (0.803) [a]	0.084 (0.915) [a]	0.122 (0.625)[a]	0.082 (0.792)
[†] R_{cryst} (%)		19.2	19.9	19.6	19.6
R_{free} (5% total data; %)		22.9	23.7	23.7	24.2
RMSD bond length[‡] (Å)		0.010 (0.019)	0.012 (0.019)	0.008 (0.019)	0.009 (0.019)
RMSD bond angles[‡] (°)		1.34 (1.96)	1.46 (1.96)	1.22 (1.96)	1.32 (1.96)
Wilson B factor[§] ($Å^2$)	40.2	30.6	31.3	34.3	36.0
Average B factor ($Å^2$)					
Protein		24.6	25.0	27.9	30.9
Solvent		39.1	39.5	42.3	44.1
Ligand		34.0	-	32.2	60

Occupancy of ligand		1.0	-	1.0	0.8
Ramachandran plot analysis (%)					
Residues in most favoured regions		98.3	97.8	98.4	98.1
Residues in outlier regions		0.26	0.13	0.13	0.13
MolProbity score [¶] [percentile]		1.75 [82nd]	1.68 [85th]	1.61 [92nd]	1.61 [92nd]
Cryoprotectant (30%)	Cryo5[#]	glycerol	Cryo5[#]	D-mannose	Cryo5[#]

Supplementary Figure 1: Size exclusion chromatography of WcbL (Figure 2)

The behavior of WcbL in size exclusion chromatography was assessed using a Superdex 200 100/30 hr column (GE Healthcare), attached to an ÄKTApurifier instrument (GE Healthcare). WcbL and standard samples were added in a volume of 100 μL, in triplicate. Elution volumes were taken as the point of highest absorbance. The standard curve was fitted to the equation:

$Log_{10}RMM = (m * \text{elution vol.}) + C$

Values for the constants m and C were determined from the data by linear regression using GraphPad Prism version 6.0.2. The standards used were dextran (2 MDa), thyroglobulin (667 kDa), apoferritin (443 kDa), alcohol dehydrogenase (150 kDa), albumin (66 kDa), and carbonic anhydrase (29 kDa). Error bars on the figure show standard error. WcbL eluted at a volume consistent with a molecular weight of 117 ± 4 kDa. As the molecular weight of each WcbL protomer is 40.53 kDa, this apparent molecular weight is most consistent with a dimer. The predicted molecular weight is less than would be expected for three protomers forming a highly spherical complex, and the crystal structure shows a dimer that deviates significantly from a sphere. Consequently, a higher hydrodynamic radius would be expected, consistent with the greater apparent molecular weight observed. Error bars represent standard error of the mean.

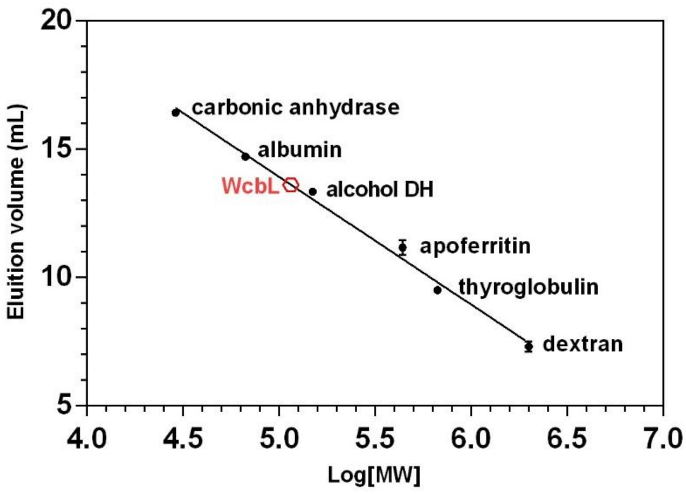

Supplementary Figure 2: Alignment of WcbL with related enzymes of known structure (Figure 3).

The three proteins with the best structural similarity to WcbL were identified using the I-TASSER server (http://zhanglab.ccmb.med.umich.edu/I-TASSER/; Yang, et al., 2015). Two of these (PDB IDs 4N3O and 3K85) are predicted to be kinases of phospho-heptoses from *Campylobacter jejuni* and *Bacteroides thetaiotaomicron* respectively. The third (PDB ID 2DEI) is a galactokinase from *Pyrococcus horikoshii*. An alignment of these three proteins was made guided by the I-TASSER alignments and edited by hand. Residues conserved between all four proteins are highlighted with a cyan background. The residues examined in this study are colored red, and highlighted with a black arrowhead. All of these are generally conserved (all orthologues have two serines in the ATP binding loop) between the four proteins with the exception of Q162 (WcbL), which is conserved in the phosphoheptose kinases, but not in the galactokinase. This is indicated with a red arrowhead.

```
2CZ9      -----MIKVKSPGRVNLIGEHTDYTY------GYVMPMAINLYTKIEAEK--HGEVILYS
4N3O      SNAMKTIRTQTPLRLGLAGGGTDINLYCDKYTGYVLNATISLYIHCTLIKREDGKIIFDS
WcbL      -MNPTIIRARAPLRLGLAGGGTDVAPYADTFGGYVLNATIDRYAYAVIKTLTIPAVRFVS
3K85      ---MSLVRSKAPLRLGLAGGGSDVSPYSDIYGGLILNATINLYAYCTIEETNSGRIEINA

2CZ9      EHFGEERKFSLNDLRKENSWIDYVKGIFWVLKESDYE---VGGIKGRVSGNLPLGAGLSS
4N3O      PDTNSYCEYESKEFLGNDGKLDIFKSIYNRIV-KDFT-KKPLSFSLHTYSDVPSGSGLGG
WcbL      TDQQVEKHQLISEPLELNGTLNLHKAVYNHMI-RNYNHGKPIALELSTFCDAPAGSGLGS
3K85      YDAQCCKSYLSMSQLEIDGEASLIKGVYNRII-RDYRL-EPKSFKITTYNDAPAGSGLGT

2CZ9      SASFEVGILETLDKLYNLKLDSLSKVLLAKKAENEFVGVPCGILDQFAVVFGREGNVIFL
4N3O      SSTLVVGVIKAFAEWLNLPLGEYEIAKLAYEIEREDLGIVGGAQDQYAATFGGFNFMEFY
WcbL      SSTLVVVMIKAFVELLNLPLDDYAIAQLAYRIERVDCGLAGGRQDQYSATFGGFNFMEFY
3K85      SSTMVVCILKAFIEWLSLPLGDYETSRLAYEIERKDLGLSGGKQDQYAAAFGGFNYMEFL

2CZ9      DTHTLDYEYIPFPK------DVSILVFYTGVRRELASSEYAERKHIAEESLKILGKGSSK
4N3O      NNKRVIVNPLRIKNWIASELEARTVLYFTNITREAKDIEEHKKG----------------
WcbL      EEERTIVNPLRIKNWVLCELEASLVLFYTGVSRESAKIIQDQSDNV--------------
3K85      QNDLVIVNPLKMKRWIVDELESSMVLYFTGRSRSSAAIINEQKKNT--------------

2CZ9      EVREGELSKLPPLHRKFFGYIVRENARVLEVRDALKEGNVEEVGKILTTAHWDLAKNYE-
4N3O      --KLGDEKSLEAMHAI---------KQDAIKMKEALFRADFGTLAQILG-KSWRSKKIISE
WcbL      --VSHKTAAIEAMHGI---------KREALVMKEALLKGDFKAFVASMR-LGWDNKKNSAR
3K85      --SEGNQTAIEAMHKI---------KQSAIDTKLALLKGDVGEFARILG-EGWENKKKMAG

2CZ9      -VSCKELDFFVERALKLGAYGARLTGAGFGGSAIALVDKEDAETIGEEILRE--YLKRFP
4N3O      IVSNDELERIYKLAIDNGAYSGKTSGAGAGGFMFFFVDPTKKYNLIKALRKEQGYVQDF-
WcbL      TVSNAHIDEIYDAAIRAGAQAGKVSGAGGGGFMLFFVPTEKRMDLIRTLGEYDGQVSNC-
3K85      AITNPMIQEAFDVATGAGAMAGKVSGAGGGGFIMFVVEPTRKEEVVRALNNLNGFVMPF-

2CZ9      WKARHFIVEPSDGVGI----------
4N3O      ----SFTKEGVKSWRI----------
WcbL      ----HFTKNGTQAWRIAN--------
3K85      ----QFIDDGAHGWKIYSTDKVQKEG
```

Supplementary Figure 3: Coordination of magnesium (Figure 3). The magnesium ion (green sphere) is shown with neighboring amino acids and AMP-PNP shown as sticks. The protein backbone is shown as cartoon, in the background. The seven atoms at reasonable distances and orientations for coordination interactions are shown as dashed lines, with the distance in Å indicated. Colors: red, oxygen; blue, nitrogen; orange, phosphorus; white, protein carbon; yellow, AMP-PNP carbon. Image produced using PyMOL.

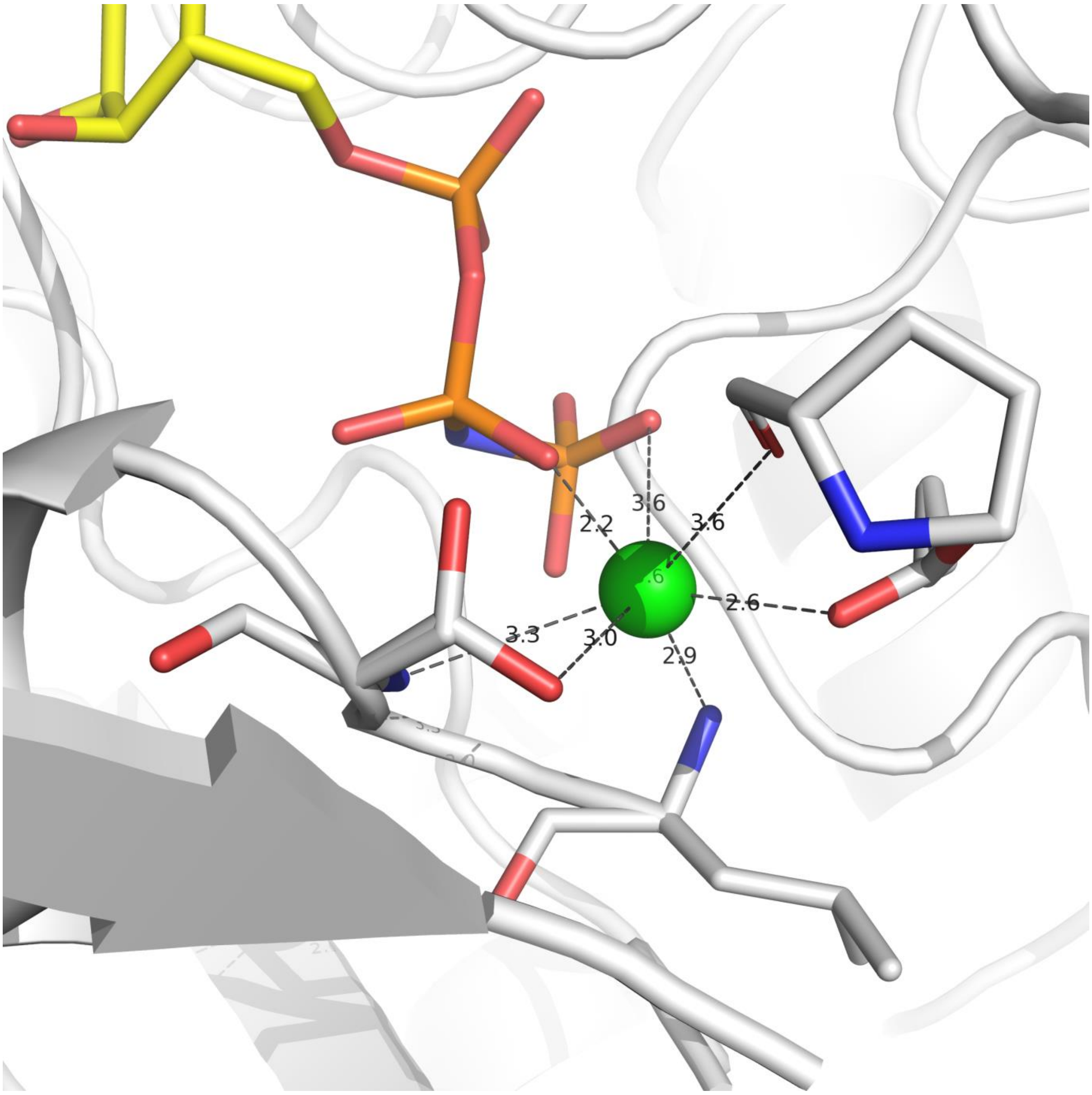

Supplementary Figure 4: Soaked glycerol occupies similar locations to soaked mannose (Figure 3). The structures of complexes with mannose (yellow carbon) and glycerol (green carbon) are shown superimposed. Protein side chains forming hydrogen bonds with either ligand are shown as sticks, and hydrogen bonds with either ligand are indicated by dashed lines, with the distance between heavy atoms in Å indicated. Colors: red, oxygen; blue, nitrogen; orange, phosphorus; purple, mannose soak protein carbon; cyan, glycerol soak protein carbon. Image produced using PyMOL.

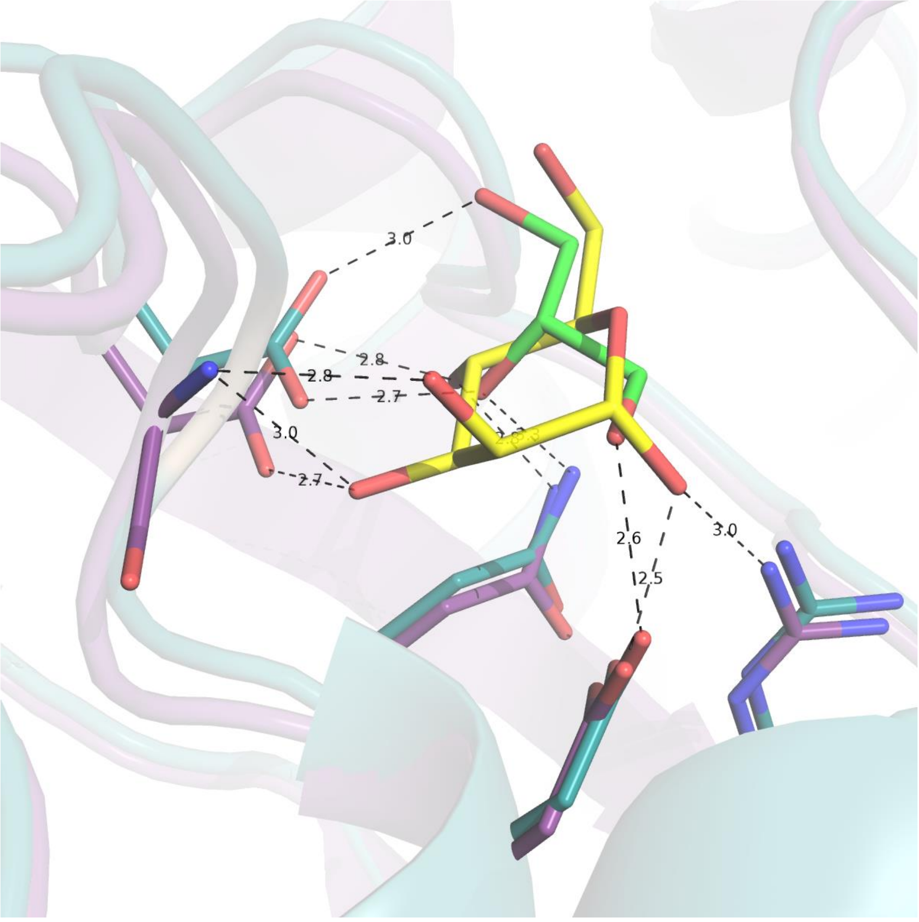

Supplementary Figure 5: Product inhibition studies reveal that WcbL uses an ordered sequential Bi-Bi reaction mechanism (Figure 4). The WcbL reaction was performed in the presence of varying amounts of the product ADP, which clearly inhibited the reaction. **A.** Reactions with differing concentrations of ATP were performed at a constant concentration of 250 μM M7P, and four concentrations of ADP. **B.** Reactions with differing concentrations of M7P were performed at a constant concentration of 1000 μM ATP, and four concentrations of ADP. **C.** The data were fit to three different inhibition models (competitive, non-competitive and uncompetitive). The fit of the best fitting model is indicated in **A** and **B**. The fits were compared using Akaike's Information Criteria, performed in GraphPad version 6.0.2. The level of certainty of the preferred model against each of the other models is indicated. Error bars represent standard error of the mean.

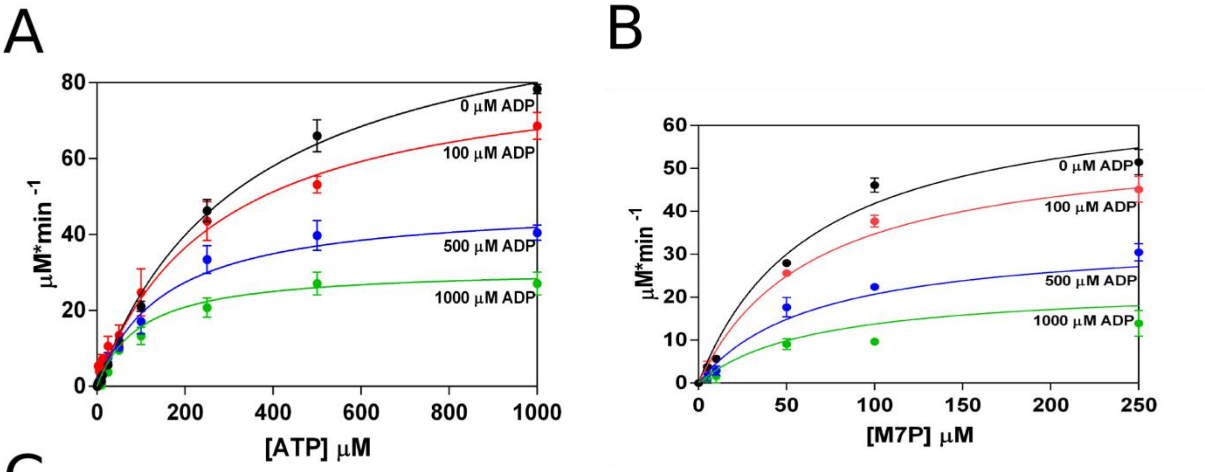

C

Varying [ATP]		Varying [M7P]	
Uncompetitive vs.:		Non-competitive vs.:	
Competitive	99.99%	Competitive	99.99%
Non-competitive	99.99%	Uncompetitive	99.98%

Supplementary Figure 6: Hydrogen bond dilution of WcbL in complex with partner compounds (Figure 5). The hydrogen-bonding network of WcbL was assessed in the absence of ligands (**A**), or the presence of one ATP molecule (**B**), two ATP molecules (**C**), two mannose molecules (**D**), two mannose and one ATP molecules (**E**) and two molecules each of mannose and ATP (**F**). Explicit hydrogens were added to the structure, and the structure optimized, using the online version of YASARA (Krieger, et al., 2009). Hydrogen bonding dilution was performed using PROFLEX (Jacobs, et al., 2001). Briefly, this method identifies hydrogen bonds from the geometry between the four atoms involved in the hydrogen bond. An energy function is then used to rank (i.e. determine the energy of) the hydrogen bonds, depending on the distances and angles between the atoms, and the chemistry of the donor and acceptor. Following this ranking, amino acids that form a network are identified. The weakest hydrogen bonds are then removed, and the networks recalculated. As the hydrogen bonds are diluted (i.e. weaker bonds removed), the networks become progressively smaller, highlighting which networks are most stable. This approach was used to highlight strongly and weakly bonded regions of the protein. The output from the hydrogen bonding dilution is shown, with sections of protein forming durable networks shown in the same color. The three main regions that bind to ATP are indicated by the red, green and blue bars to the left of each image.

In the ligand free state (**A**), both protomers show equivalent rigidity. The rigidity of the protein is increased with the binding of one ATP molecule (**B**: ~0.2 kcal/mol greater stability of the bound protomer) or two mannose molecules (**D**: ~0.1 kcal/mol greater stability compared to the one ATP bound form). This rigidity is lost upon binding of a second ATP (**C**: ~0.3 kcal/mol less stable than the *apo*-structure). A similar effect is observed upon binding one or two ATP molecules to the mannose-bound structure (**E, F**): little difference is seen on binding the second ATP when mannose is bound.

A: Native

Wed Feb 4 13:04:13 2015

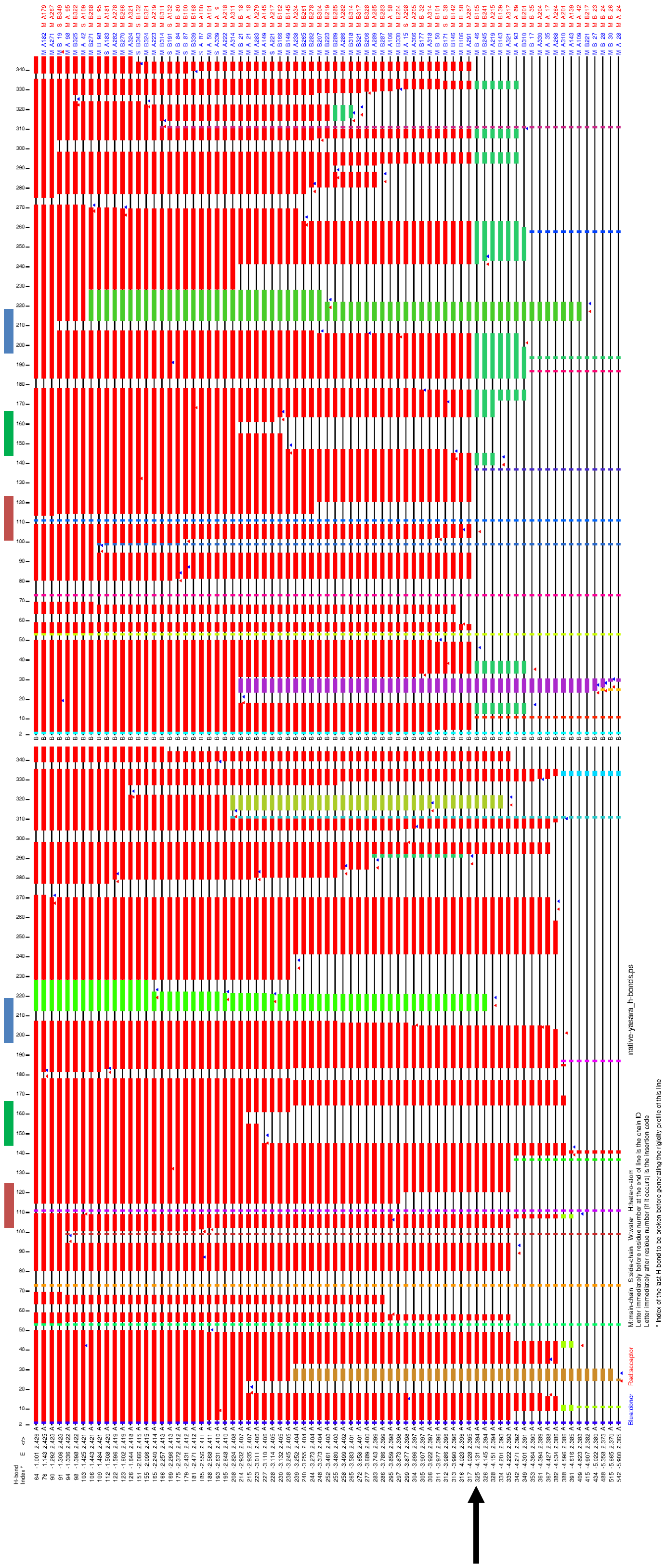

B: 1 ATP (molecule 1)

Wed Feb 4 13:13:33 2015

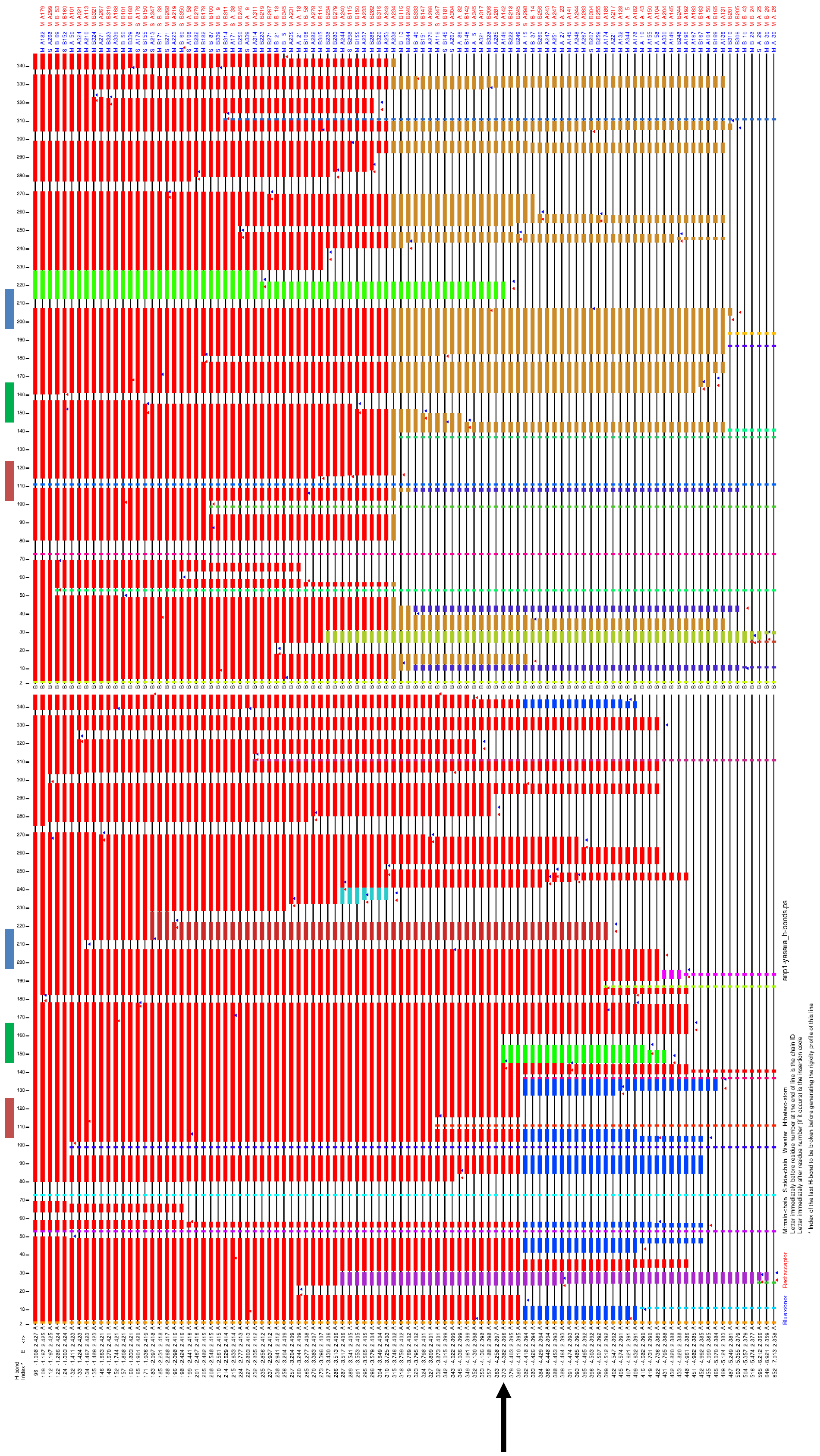

C: 2 ATP

Wed Feb 4 15:42:36 2015

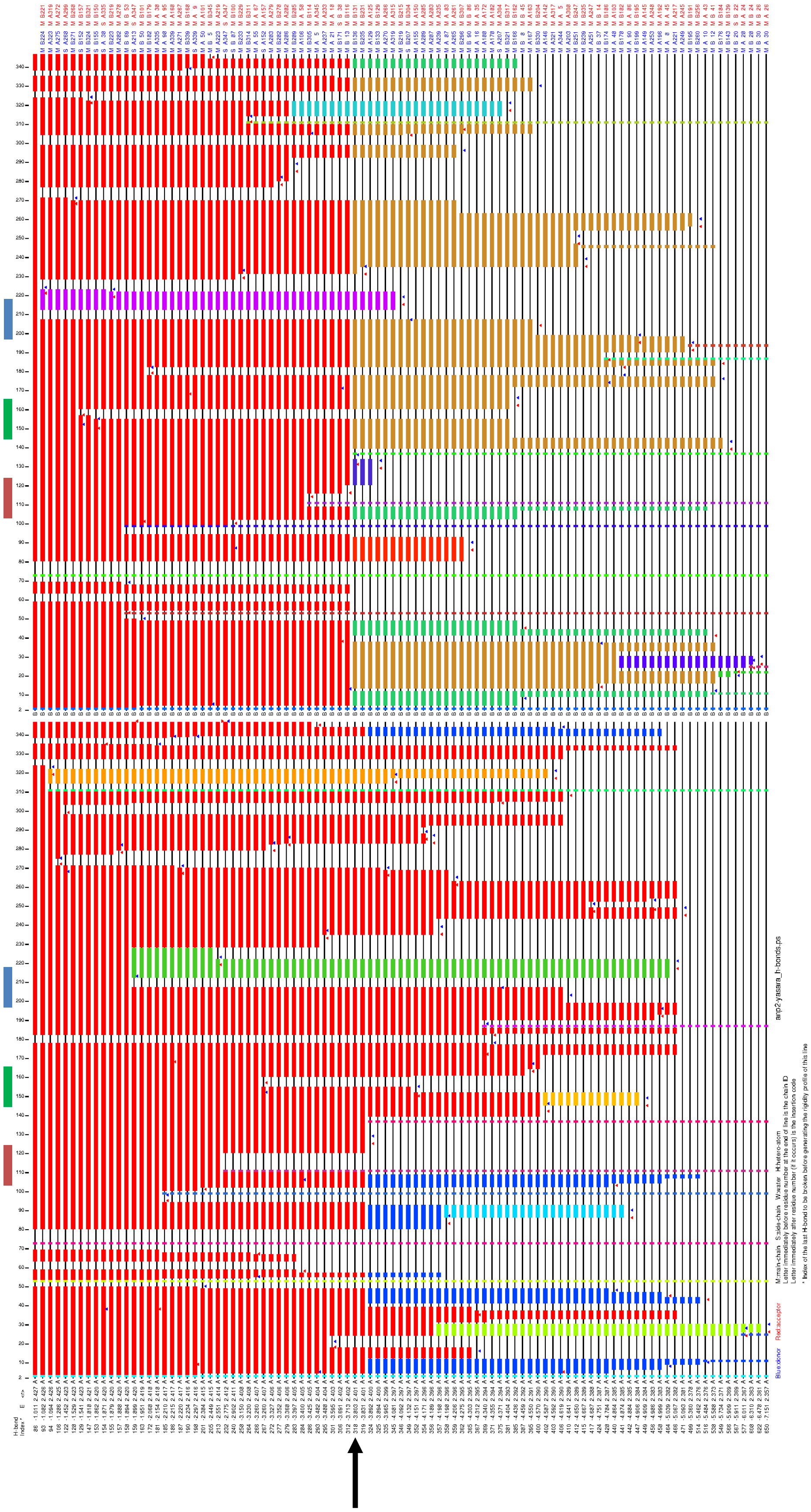

D: 2 mannose

Wed Feb 4 15:59:03 2015

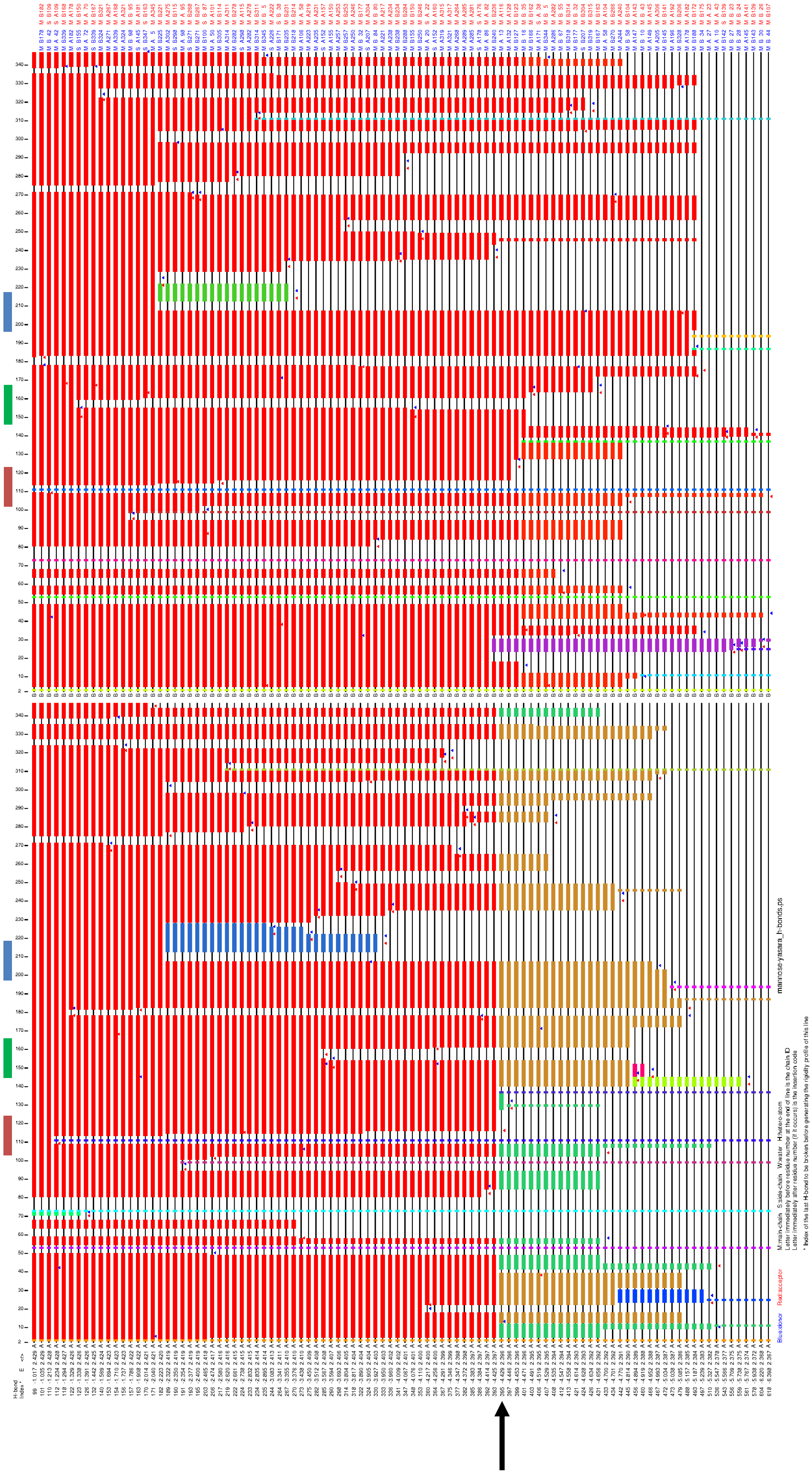

E: 1 ATP (molecule 2) and 2 mannose:

Wed Mar 4 10:30:01 2015

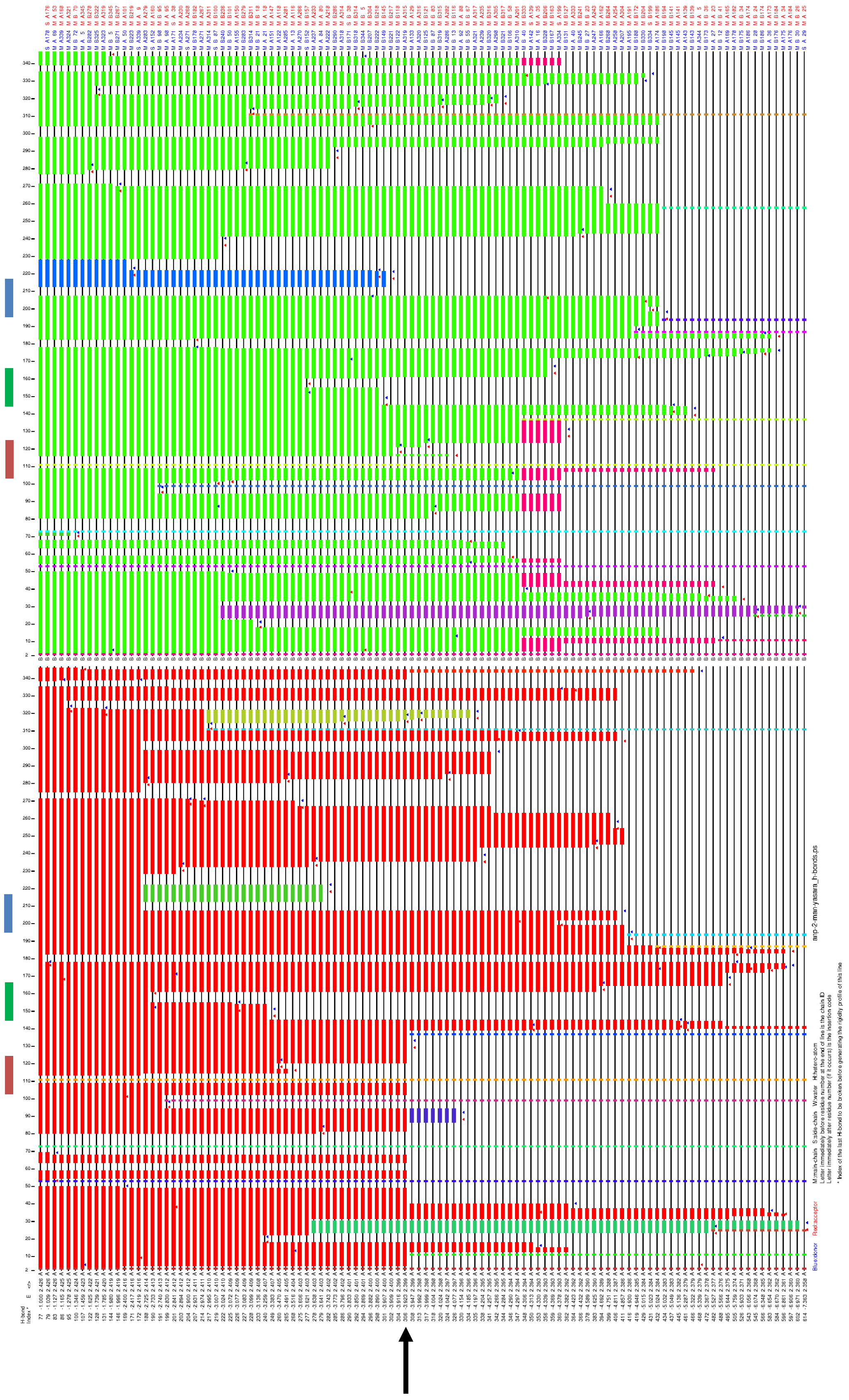

F: 2 ATP and 2 mannose

Wed Feb 4 16:07:42 2015

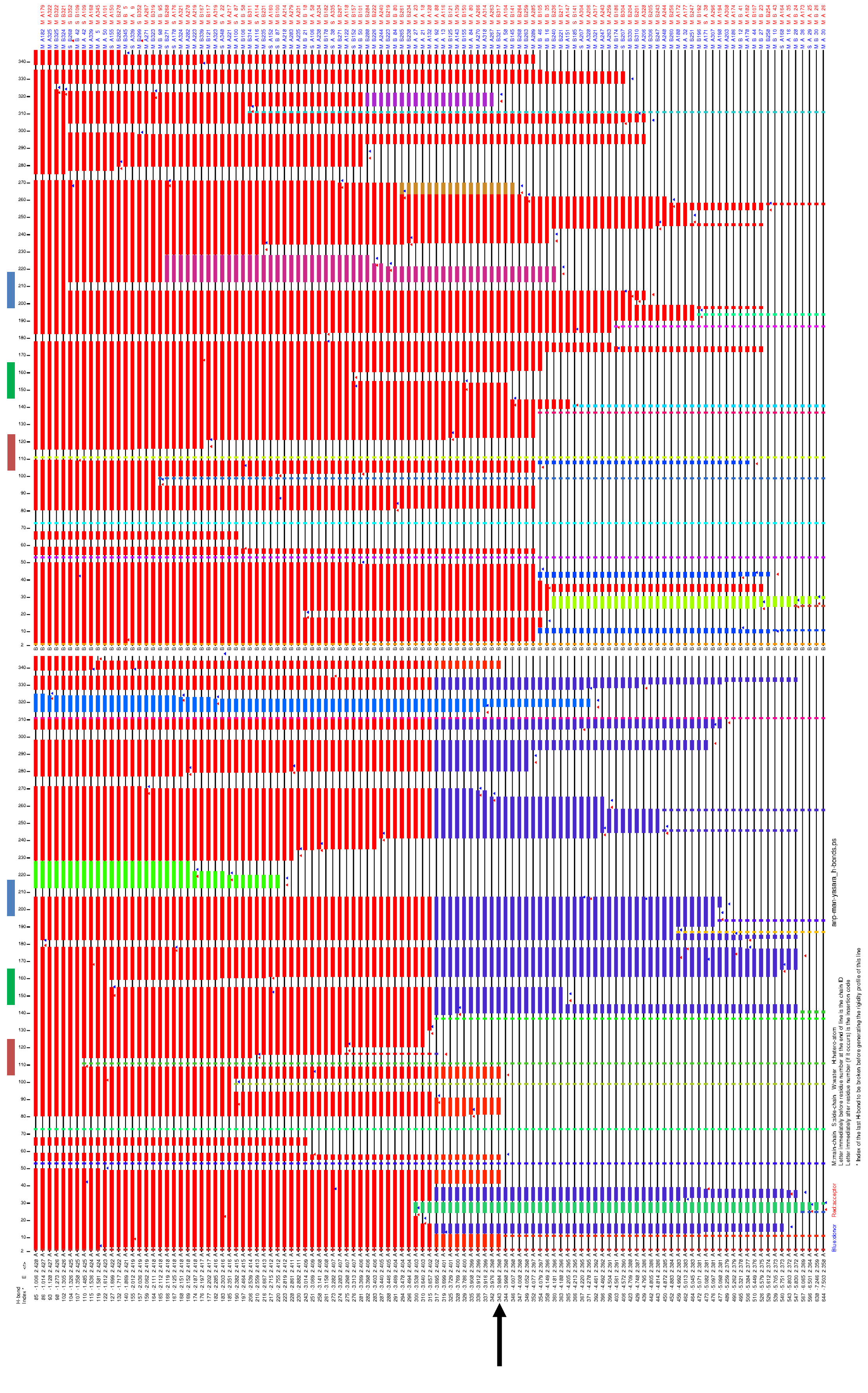

Supplementary references

Chen, V.B., Arendall, W.B., 3rd, Headd, J.J., Keedy, D.A., Immormino, R.M., Kapral, G.J., Murray, L.W., Richardson, J.S., and Richardson, D.C. (2010). MolProbity: all-atom structure validation for macromolecular crystallography. Acta Crystallogr D Biol Crystallogr 66, 12-21.

Jacobs, D.J., Rader, A.J., Kuhn, L.A., and Thorpe, M.F. (2001). Protein flexibility predictions using graph theory. Proteins 44, 150-165.

Krieger, E., Joo, K., Lee, J., Lee, J., Raman, S., Thompson, J., Tyka, M., Baker, D., and Karplus, K. (2009). Improving physical realism, stereochemistry, and side-chain accuracy in homology modeling: Four approaches that performed well in CASP8. Proteins 77, 114-122.

Vaguine, A.A., Richelle, J., and Wodak, S.J. (1999). SFCHECK: a unified set of procedures for evaluating the quality of macromolecular structure-factor data and their agreement with the atomic model. Acta Crystallogr D Biol Crystallogr 55, 191-205.

Yang, J., Yan, R., Roy, A., Xu, D., Poisson, J., and Zhang, Y. (2015). The I-TASSER Suite: protein structure and function prediction. Nat Methods 12, 7-8.
