# Supplementary material for: Unraveling the B. pseudomallei Heptokinase WcbL: From Structure to Drug Discovery
Source: Chem Biol. 2015 Dec 17;22(12):1622–32. doi: 10.1016/j.chembiol.2015.10.015 (PMC4691232; doi:10.1016/j.chembiol.2015.10.015)
Supplement: Document S2. Article plus Supplemental Information [file mmc2.pdf]

Article

Chemistry & Biology

Unraveling the *B. pseudomallei* Heptokinase WcbL: From Structure to Drug Discovery

Graphical Abstract

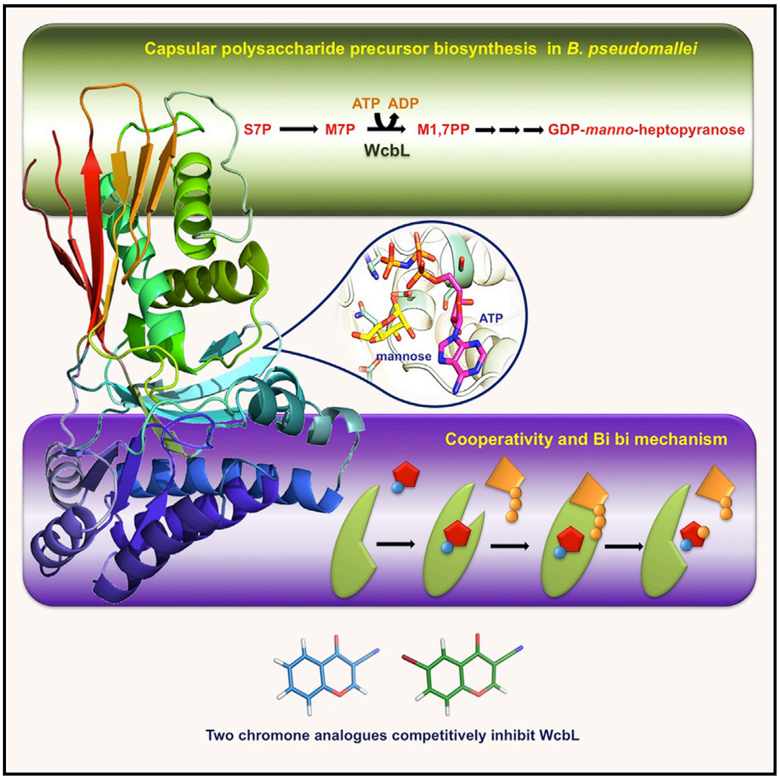

Authors

Mirella Vivoli, Michail N. Isupov, Rebecca Nicholas, ..., Paul Kosma, Joann L. Prior, Nicholas J. Harmer

Correspondence

n.j.harmer@exeter.ac.uk

In Brief

Vivoli et al. present the structure and mechanism of the bacterial sugar kinase WcbL. WcbL demonstrates unusual kinetics: its cooperativity for ATP switches on binding of the sugar substrate. Screening of a fragment library identified a potent inhibitor of WcbL.

Highlights

- The structure of WcbL in complex with ligand analogs is reported
- WcbL switches its cooperativity toward ATP in the presence of sugars
- The kinetics and mechanism of WcbL are elucidated
- Screening of a fragment library identified a potent inhibitor of WcbL

Accession Numbers

4USK
4UTG
4USM
4UT4

Vivoli et al., 2015, Chemistry & Biology *22*, 1622–1632
December 17, 2015 ©2015 The Authors
http://dx.doi.org/10.1016/j.chembiol.2015.10.015

Unraveling the *B. pseudomallei* Heptokinase WcbL: From Structure to Drug Discovery

Mirella Vivoli,[1] Michail N. Isupov,[1] Rebecca Nicholas,[1] Andrew Hill,[1,4] Andrew E. Scott,[2] Paul Kosma,[3] Joann L. Prior,[2] and Nicholas J. Harmer[1,*]
[1]Department of Biosciences, University of Exeter, Henry Wellcome Building, Stocker Road, Exeter EX4 4QD, UK
[2]Defence Science and Technology Laboratory, Porton Down, Salisbury, Wiltshire SP4 0JQ, UK
[3]University of Natural Resources and Life Sciences-Vienna, Muthgasse 18, 1190 Vienna, Austria
[4]Present address: School of Chemistry, University of Manchester, Oxford Road, Manchester M13 9PL, UK
*Correspondence: n.j.harmer@exeter.ac.uk
http://dx.doi.org/10.1016/j.chembiol.2015.10.015
This is an open access article under the CC BY license (http://creativecommons.org/licenses/by/4.0/).

SUMMARY

Gram-negative bacteria utilize heptoses as part of their repertoire of extracellular polysaccharide virulence determinants. Disruption of heptose biosynthesis offers an attractive target for novel antimicrobials. A critical step in the synthesis of heptoses is their 1-*O* phosphorylation, mediated by kinases such as HldE or WcbL. Here, we present the structure of WcbL from *Burkholderia pseudomallei*. We report that WcbL operates through a sequential ordered Bi-Bi mechanism, loading the heptose first and then ATP. We show that dimeric WcbL binds ATP anticooperatively in the absence of heptose, and cooperatively in its presence. Modeling of WcbL suggests that heptose binding causes an elegant switch in the hydrogen-bonding network, facilitating the binding of a second ATP molecule. Finally, we screened a library of drug-like fragments, identifying hits that potently inhibit WcbL. Our results provide a novel mechanism for control of substrate binding and emphasize WcbL as an attractive anti-microbial target for Gram-negative bacteria.

INTRODUCTION

Bacterial cells display a variety of polysaccharides and glycoconjugates in their cell walls and external structures. The functions of these macromolecules include maintaining the structural integrity of the cell (e.g. peptidoglycan), defense against a diverse range of environmental insults, and providing virulence determinants for the invasion of hosts (Lovering et al., 2012; Matsuura, 2013; Whitfield, 2006). The polysaccharides of particular relevance for infection are the lipopolysaccharides (LPS) and capsular polysaccharides (CPS). The LPS core is required in most bacteria for the structural integrity of the outer membrane (Raetz and Whitfield, 2002), while LPS O antigens and CPS often provide bacteria with protection in different or novel niches, including the human body (Kingsley and Baumler, 2000; Whitfield, 2006). These extracellular structures surrounding the microbial cell consist of polymers of sugars that are often not found in eukarya.

One important group of bacterial sugars are the heptoses (Kosma, 2008). These are found in the LPS core of most common bacteria (Figure 1A) and in many O antigens or CPS (e.g. *Shigella*, *Yersinia pseudotuberculosis*, *Campylobacter jejuni* O antigen; *Burkholderia pseudomallei* CPS-I [DeShazer et al., 2001; Pacinelli et al., 2002; Reckseidler et al., 2001]). Depending on the final product that is required by the bacterium, these sugars are biosynthesized from the ubiquitous pentose phosphate pathway intermediate sedoheptulose-7-phosphate (S7P) by one of two biological pathways (Butty et al., 2009). For α-heptopyranosides, which generally occur in the LPS core, D-*glycero*-D-*manno*-heptopyranose is synthesized from S7P as a β-configured ADP conjugate in a four-step pathway. Two steps are catalyzed by a bifunctional enzyme, HldE. β-D-*manno*-Heptopyranosides, usually being present in O antigens or CPS, are similarly synthesized as an α-guanosine diphosphate (GDP) conjugate via a four-step pathway: two enzymes, GmhA and GmhB, are shared between the two pathways, but HldE is replaced by two separate enzymes. In some organisms, the GDP-heptopyranose is then further processed to the 6-deoxy sugar (e.g. *C. jejuni*, *Y. pseudotuberculosis*; Figure 1B) (Gilbert et al., 2007; Pacinelli et al., 2002).

The biosynthetic pathways for heptoses have been proposed as an excellent source of novel antibiotics (Durka et al., 2011; Taylor and Wright, 2008): similar sugars are not found in mammals, and the sugars are widely used by bacteria, so any inhibitor would have a broad spectrum. Compounds active against the kinase activity of HldE at micromolar concentrations have been identified (De Leon et al., 2006; Lee et al., 2013). The development of compounds targeting the GDP-linked biosynthetic pathways has not had the same attention, partly because the kinases have not been as thoroughly characterized as HldE. These GDP-linked pathways are usually used in O antigens and CPS, which are usually required for infectivity, not viability. As such, antibiotics are likely to have lower selection pressure for resistance, and fewer effects on the commensal flora.

The Gram-negative bacterium *B. pseudomallei* requires CPS for full virulence (Atkins et al., 2002; Nelson et al., 2004; Vivoli et al., 2014a). This pathogen is the causative agent of melioidosis, the most common cause of community-acquired septicemia in many regions of South-East Asia (Stone, 2007; Wiersinga

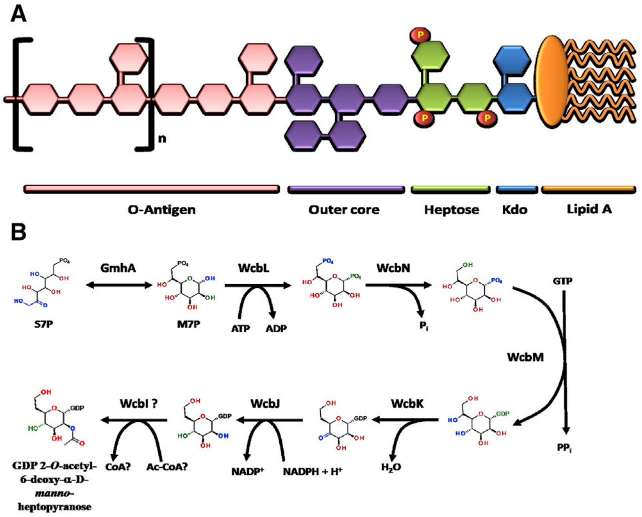

Figure 1. Heptoses in Gram-Negative Bacteria

(A) Schematic representation of exopolysaccharide structures. Lipopolysaccharide (LPS) forms the outer leaflet of the outer membrane of Gram-negative bacteria, which comprises several regions: the lipid A portion composed of two linked glucosamine residues (oblongs) with fatty acid side chains (curved lines), an internal oligosaccharide bound to lipid A by 3-deoxy-D-manno-oct-2-ulosonic acid (Kdo; blue hexagon). Heptose moieties (green hexagons; P, phosphate groups) complete the inner core of LPS. Attached to the inner core are various hexose sugars (violet hexagons), which form the outer core region. The O chain is a repeating unit (n) of specific sugar residues (pink hexagons) that forms the basis for serotyping, and is bound to the internal oligosaccharide. The capsular polysaccharide (CPS), associated with the LPS, is an unbranched homopolymer with the structure -3)-2-*O*-acetyl-6-deoxy-β-D-*manno*-heptopyranose-(1-.

(B) Proposed biosynthetic pathway of CPS in *Burkholderia pseudomallei*. GDP, guanosine diphosphate; GTP, guanosine triphosphate; PP_i, pyrophosphate.

et al., 2012). This organism is a major clinical problem as it shows intrinsic high-level resistance to many antibiotics, and relapse is common even after long-term antibiotic treatment (Lazar Adler et al., 2009). Mortality from septicemia ranges from 14% to 50%, depending on treatment, and up to 90% if untreated (Limmathurotsakul and Peacock, 2011; White, 2003). The organism is also considered to be a significant threat for bioterrorism (Rotz et al., 2002) and is currently classed by the US Centers for Disease Control and Prevention as a tier-1 agent (Butler, 2012); new treatments are being urgently sought by the USA and UK defense agencies. In *B. pseudomallei*, CPS is biosynthesized by a cluster of 24 genes (DeShazer et al., 2001; Holden et al., 2004; Reckseidler et al., 2001), whose function has been proposed from bioinformatics and functional studies (Cuccui et al., 2012). In the initial steps of CPS production, GmhA transforms S7P into D-*glycero*-D-*manno*-heptose-7-phosphate, which is further phosphorylated. It has been proposed that the heptokinase activity is contributed by WcbL (Cuccui et al., 2012). We therefore sought to determine the structure and mechanism of the WcbL protein, and to understand how amenable it might be to being inhibited by a novel compound.

Here, we describe the crystal structure of WcbL, solved to 1.78 Å, together with separate complexes with the substrate analogs AMP-PNP and D-mannose. The structure shows a classical GHMP sugar kinase fold, and maintains some of the conserved residues required for ATP binding and catalysis. Combining the positions of AMP-PNP and mannose suggests that these analogs occupy the likely positions of the true substrates before catalysis commences. We demonstrate that mutation of likely active-site residues ablates enzyme activity both in vitro and in vivo, allowing us to propose a catalytic mechanism. We show that WcbL modulates its cooperative behavior for ATP depending on whether it has its sugar partner bound, and that this is mediated through the global hydrogen-bonding network. Finally, we show that specific inhibition of WcbL by drug-like fragments can be achieved with high ligand efficiency, indicating that the development of high-affinity, specific inhibitors of the GDP-linked heptose pathway should be possible.

RESULTS

Overall Structure of WcbL

To improve our understanding of WcbL, we solved the crystal structure of the enzyme to 1.78 Å resolution (Table S1). The structure was solved using the single-wavelength anomalous dispersion (SAD) method, using selenomethionine-labeled protein to provide a strong anomalous signal. WcbL crystals seem to have lattice translocation disorder, as can be judged by streaky reflections in certain directions. Crystals appear to contain domains with the orthorhombic base-centered unit cell and with a smaller monoclinic base-centered cell in which pseudotranslation observed in the orthorhombic cell becomes crystallographic. Native WcbL and an AMP-PNP complex had a lower proportion of the monoclinic fraction: for these complexes data did not process well and the model did not refine in the monoclinic space group. The glycerol and mannose complex crystals had a higher proportion of the monoclinic cell: these data could be processed, and the models refined well in both space groups. However, ligand electron density was significantly better in the orthorhombic space group for both of these complexes, and therefore all structures were refined in the orthorhombic cell. Native crystals formed in a number of related space groups in identical conditions, and determining the correct space group was challenging for many crystals. The solved structure reveals that WcbL adopts, as expected, the GHMP sugar kinase fold (Figure 2A). A similar fold is seen in the structure of a human *N*-acetylgalactosamine kinase (PDB: 2A2C; Figure 2B). The protein forms a head-to-tail dimer, with the two active sites facing away from the dimer interface (Figure 2C), in line with the native molecular weight predicted from size-exclusion chromatography (Figure S1). A similar molecular arrangement is seen in the structures of two putative sugar kinases from *Bacteroides thetaiotamicron* and *C. jejuni* (PDB: 3K85 and 4N3O, respectively), which share 41% sequence identity with WcbL.

Examination of the active site shows an enlarged binding pocket, compared with the highest sequence identity homolog with a solved structure (a galactokinase, PDB: 2DEI). Analysis of these structures with CAVER v.3.0 (Chovancova et al., 2012) showed a 27% increase in the pocket size, consistent with the larger sugar substrate M7P. Comparison of the sequence with galactokinases reveals a Q-L mutation at position 162 (Figure S2). This facilitates the accommodation of the

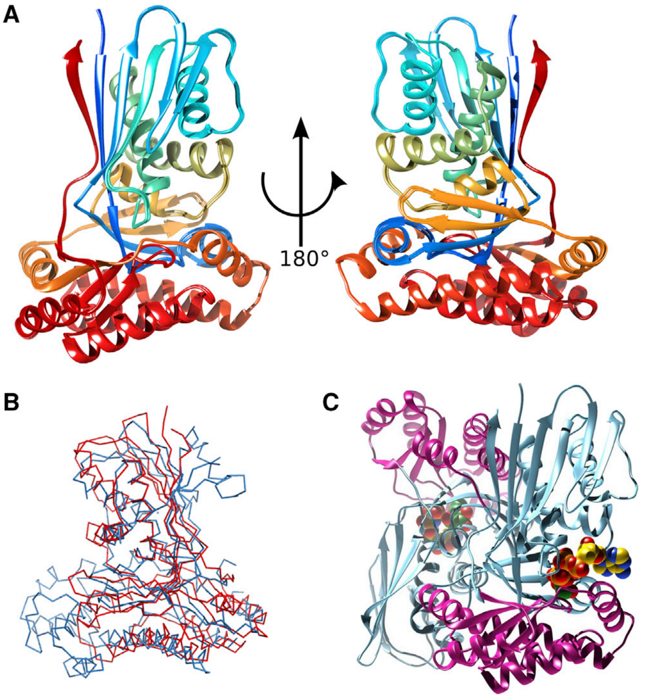

Figure 2. Overview of WcbL Structure

(A) Monomeric crystal structure of WcbL in two orientations, showing the GHMP kinase fold. The backbone is shown in cartoon representation, rainbow colored from red (N terminus) to blue (C terminus).

(B) Superimposition of a representative sugar kinase onto the structure of WcbL. The structures are shown as ribbons: WcbL, red; human *N*-acetylgalactosamine kinase (PDB: 2A2C), blue.

(C) Head-to-tail dimer of WcbL, with the two active sites facing away from the dimer interface. The two domains of WcbL are colored cyan and magenta. AMP-PNP and mannose are shown as spheres. Colors: nitrogen, blue; oxygen, red; phosphorus, orange; AMP-PNP carbon, yellow; mannose carbon, green.

Images (A) and (C) were prepared using the UCSF Chimera package from the Computer Graphics Laboratory, University of California, San Francisco. (B) was prepared with the PyMOL Molecular Graphics System (Schrödinger).

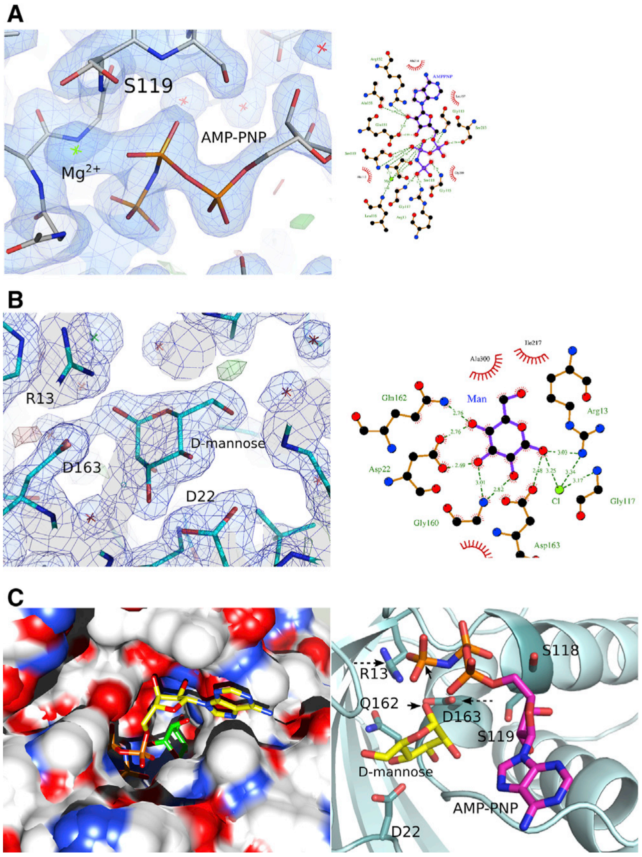

Figure 3. Interaction of WcbL with Substrates

(A) AMP-PNP binding to WcbL. Electron density map showing the binding of AMP-PNP to WcbL with both molecules as sticks (left). The Mg^{2+} is shown in green; co-ordination of the magnesium is shown in Figure S3. The $2F_o - F_c$ electron density map (blue) was contoured at 1.06σ for the AMP-PNP and the neighboring residues; the positive and the negative $2F_o - F_c$ electron density maps were both contoured at 3σ. To the right is a schematic drawing of the WcbL-AMP-PNP interactions.

(B) Binding of D-mannose to WcbL. Electron density map showing the binding of the sugar to the protein; both molecules are shown as sticks. The $2F_o - F_c$ electron density map (blue) was contoured at 1.3σ for the D-mannose and the neighboring residues; the positive and the negative $2F_o - F_c$ electron density maps were both contoured at 3σ (left); key interactions between WcbL and D-mannose are shown on the right. The electron density map figures were prepared with the PyMOL Molecular Graphics System (Schrödinger), and the schemes of interactions between WcbL and the substrates were prepared with LigPlot+.

(C) Electrostatic surface representation of WcbL, showing AMP-PNP (yellow and orange sticks) and D-mannose (green sticks) in the binding pocket (figure produced using the UCSF Chimera package, Computer Graphics Laboratory, University of California, San Francisco). WcbL, AMP-PNP, and D-mannose interactions are shown on the right. WcbL is shown as a cyan cartoon, with interacting side chains as sticks; AMP-PNP and D-mannose are shown as sticks. AMP-PNP: carbon, violet; nitrogen, blue; oxygen, red; phosphorus, orange; D-mannose carbon, yellow. WcbL: nitrogen, blue; oxygen, red. Atoms involved in the catalytic reaction are indicated by black arrowheads; key catalytic residues R13 and D163 are indicated by dashed arrows. Figure produced using PyMOL.

extra carbon unit in the heptose sugar compared with the hexose substrate of the related enzymes. Repeated attempts to soak or co-crystallize the substrates M7P and ATP failed. However, soaking with the non-hydrolyzable substrate analog AMP-PNP showed clear density for this nucleotide (Figure 3A). Furthermore, soaking of native crystals with glycerol or mannose showed that these two sugars both bind in the sugar-phosphate binding pocket, where the sugar is expected (Figure 3B; Figure S4). Superimposing the locations of AMP-PNP and mannose shows that the location of these molecules is compatible (Figure 3C). Indeed, the reactive atoms (O_1 of mannose, P_γ of AMP-PNP) are located in positions compatible with the expected reaction (nucleophilic attack of O_1 on the γ-phosphate). This suggests that the locations of these two substrate analogs in the structure are representative of the true ligands. The ATP analog appears to form a network of interactions with the enzyme, with the majority of interactions formed with the phosphate, and the ribose O2 and O3 hydroxyls. The phosphates interact with the side chains of R13 and D163, while the ribose ring interacts with the side chains of S118 and S119. Interactions of the sugar substrate analog principally form with the side chains of D22, Q162, and D163.

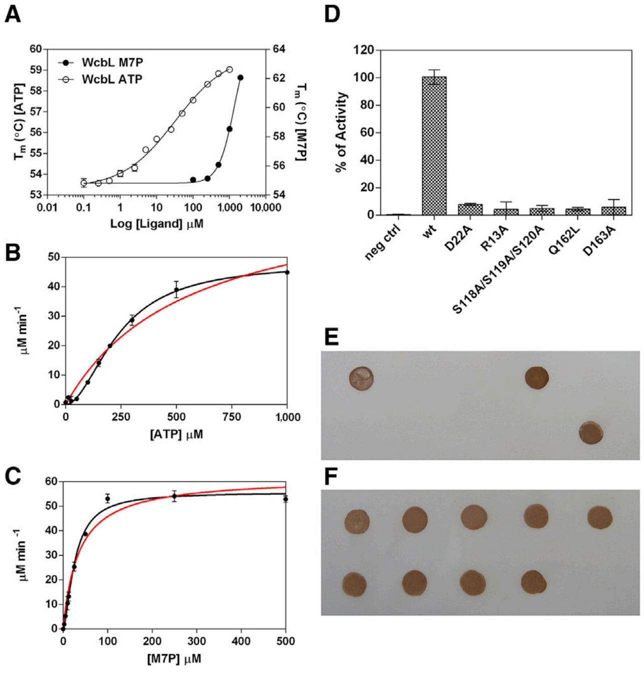

Figure 4. Kinase Activity and Cooperativity of WcbL

(A) Biophysical characterization of WcbL binding to ATP reveals an anti-cooperative binding. The apparent dissociation constant K_D is 33 ± 7 μM with the cooperativity coefficient n = 0.6 ± 0.06, indicating a negative cooperativity (open circles). The DSF experiment for M7P binding (closed circles) gives an apparent dissociation constant K_D for M7P of 968 ± 37 μM. All experiments were performed in triplicate, and the data are representative of at least three experiments. We separately showed that ATP shows negligible degradation at 58°C during the relevant length of this experiment (not shown).

(B and C) WcbL kinase activity. For comparison, data were fitted to both the Michaelis-Menten equation (red line) and an allosteric sigmoidal model (black line).

(B) Wild-type WcbL activity is plotted as a function of ATP concentration, keeping the concentration of M7P constant at 250 μM. An apparent k_{cat} value of 157 ± 5 min^{-1} was calculated for wild-type. Calculated $K_{1/2}$ value for ATP was 240 ± 12 μM, whereas the Hill slope value h was 1.9 ± 0.1, suggesting positive cooperativity.

(C) Allosteric behavior is observed for WcbL wild-type with the substrate M7P, maintaining the concentration of ATP at 1 mM. Calculated $K_{1/2}$ and k_{cat} values for the M7P substrate were 27 ± 1.6 μM and 182 ± 4 min^{-1}, respectively, whereas the Hill coefficient was 1.5 ± 0.1.

(D) WcbL activity of wild-type and mutants. The kinetic assays were performed using 6 μg/ml of WcbL wild-type and 60 μg/ml of mutants, keeping the concentrations of ATP and M7P constant at 1 mM and 0.5 mM, respectively. All experiments were performed in triplicate, and the data are representative of at least three experiments. Negative control represents the experiment carried out without WcbL. Error bars represent SEM.

(E) In vivo validation of key ligand binding and catalytic residues. Western hybridization using the capsule-specific monoclonal antibody 4VIH12 demonstrates restoration of capsule production in *B. pseudomallei* K96243 Δ*wcbL* when complementation is with unaltered *wcbL*. Complementation with *wcbL* site-directed mutants fails to restore capsule production.

(F) Western hybridization using the O antigen-specific monoclonal antibody CC6 demonstrates continued expression of native lipopolysaccharide in all strains tested. No LPS is seen in the purified capsule control (10). In each panel strains are spotted in the order: 1, *B. pseudomallei* K96243; 2, *B. pseudomallei* K92643 Δ*wcbL*; 3, *B. pseudomallei* K92643 Δ*wcbL* (pBBR1-MCS2); 4, *B. pseudomallei* K92643 Δ*wcbL* (pBBR1-MCS2-*wcbL*); 5, *B. pseudomallei* K92643 Δ*wcbL* (pBBR1-MCS2-*wcbL* S118A/S119A/S120A); 6, *B. pseudomallei* K92643 Δ*wcbL* (pBBR1-MCS2-*wcbL* R13A); 7, *B. pseudomallei* K92643 Δ*wcbL* (pBBR1-MCS2-*wcbL* D22A); 8, *B. pseudomallei* K92643 Δ*wcbL* (pBBR1-MCS2-*wcbL* Q162L); 9, *B. pseudomallei* K92643 Δ*wcbL* (pBBR1-MCS2-*wcbL* D163A); 10, purified capsule from *B. pseudomallei* K96243.

ATP and M7P Binding Study by Differential Scanning Fluorimetry

To further understand the activity of WcbL, we investigated its binding to ATP using differential scanning fluorimetry (DSF) (Niesen et al., 2007; Vivoli et al., 2014b). These data (Figure 4A) showed an apparent negative cooperativity of binding: the data suggested a dissociation constant $K_{1/2}$ (the concentration of substrate that produces a half-maximal enzyme velocity) of 33 ± 7 μM with the cooperativity coefficient n = 0.6 ± 0.06, indicating an apparent negative cooperativity. As WcbL is dimeric, this implies that the enzyme is perfectly anti-cooperative in its binding to ATP. On the other hand, M7P did not show any cooperativity, and a dissociation constant of 968 ± 37 μM.

WcbL Activity and Kinetic Mechanism

WcbL activity was predicted due to its high sequence similarity to HddA from *Y. pseudotuberculosis* (Cucci et al., 2012; Pacinelli et al., 2002). This is thought to catalyze the phosphorylation of D-α,β-D-heptose-7-phosphate to D-α-D-heptose-1,7PP (M7PP) using ATP. To investigate the kinase function of WcbL, we performed a discontinuous coupled assay, based on the concerted action of WcbL and WcbN with detection of the released inorganic phosphate. The kinetic data for ATP fitted best to a cooperative model. $K_{1/2}$ and k_{cat} for ATP were determined as 240 ± 12 μM and 157 ± 5 min^{-1}, respectively, with a Hill coefficient value h of 1.9 ± 0.1 (Figure 4B). Despite the negative cooperativity observed upon ATP binding in the DSF experiment, the kinetic data suggested positive cooperativity. The kinetic parameters for M7P also fitted best to a cooperative model. The $K_{1/2}$, k_{cat}, and h values were determined to be 27 ± 1.6 μM, 182 ± 4 min^{-1}, and 1.5 ± 0.1, respectively (Figure 4C).

The structure of WcbL bound to AMP-PNP suggests that the ATP may bind in a manner that blocks access to the sugar-phosphate binding site. We therefore investigated the likely reaction kinetics further by product inhibition experiments using ADP as inhibitor. Addition of ADP caused changes in both K_M and V_{max}. The kinetic data varying ATP concentrations shows a non-competitive inhibition which becomes uncompetitive at high concentrations of ADP (K_i = 411 ± 27 μM), whereas ADP inhibition at different concentrations of M7P is non-competitive (K_i = 490 ± 41 μM; Figure S5). These results strongly suggest an ordered sequential Bi-Bi mechanism of reaction. This is distinguished from a random sequential Bi-Bi reaction (where ADP is expected to act as a competitive inhibitor for ATP) and a ping-pong mechanism (where ADP is expected to be a competitive inhibitor of M7P). The structural and kinetic data are therefore both consistent with an ordered sequential Bi-Bi reaction mechanism whereby M7P binds to WcbL prior to ATP, and ADP releases prior to D-*manno*-α-D-heptose-1,7-bisphosphate.

Mutation of Key Ligand Binding and Catalytic Residues Reveal a Likely Mechanism for Enzyme Activity

To better understand the action of the enzyme, we made mutants in a range of likely substrate binding and catalytic residues.

A

Ligand	Minimum hydrogen bond energy of major structural elements (kcal/mol)
None	4.1
1 ATP	4.3
2 ATP	3.8
2 mannose	4.4
2 mannose, 1 ATP	3.9
2 mannose, 2 ATP	4.0

B

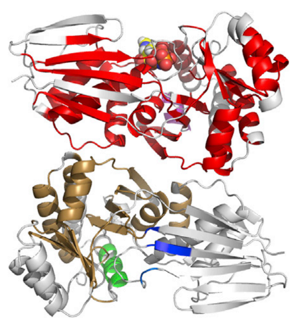

C **D**

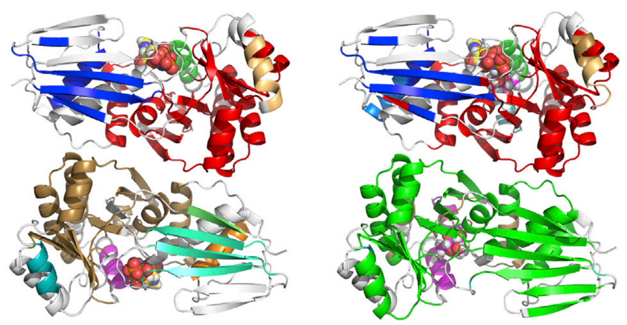

Figure 5. WcbL Shows Differential Cooperativity for ATP when Mannose Bound

(A) Table indicating the hydrogen-bond energies where the hydrogen-bonding network starts to collapse.

(B–D) Analysis of the hydrogen-bonding network of WcbL reveals an anti-cooperative effect in the absence of the sugar substrate. The assessed hydrogen-bonding network of WcbL is shown in the presence of one ATP molecule (B), two ATP molecules (C), and two molecules each of mannose and ATP (D). Hydrogen-bonding dilution was performed using Proflex (Jacobs et al., 2001). Groups of residues in each structure that form part of a hydrogen-bonding network that persists when hydrogen bonds weaker than 3.9 kcal/mol (the point where the network ruptures in D) are removed are indicated by different colors (colors match those in Figure S6). Bound ligands are shown as all heavy atom representations (nitrogen, blue; oxygen, red; phosphorus, orange; ATP carbon, yellow; mannose carbon, magenta). More detailed analysis of the hydrogen-bonding network is shown in Figure S6.

The residues chosen for mutation were residues involved in heptose binding (Q162L and D22A), heptose/phosphate binding (D163A), and ATP binding (R13A and S118A/S119A/S120A triple mutant). All of these mutant proteins purified like the wild-type. Every mutant led to a complete loss of catalytic activity (Figure 4D). In each case, the activity was below the limit of detection of the assay, and at least 150 times less than the wild-type. To further demonstrate the loss of function of these mutants, we complemented the previously described *B. pseudomallei wcbL*$^{-}$ mutant (Cuccui et al., 2007) with either wild-type WcbL or with one of the mutants. While wild-type enzyme successfully complemented the mutant and restored production of CPS-I (Figures 4E and 4F), none of the mutants was able to restore any function. These observations strongly support the notion that the interactions with ATP and M7P predicted from the crystal structures represent the biologically relevant binding of these substrates.

To further probe the mechanism, we performed an in silico examination of the normal modes and hydrogen-bonding networks of WcbL in the presence and absence of these ligands. These analyses suggested that there was no significant alteration in the major normal modes of the enzyme in the presence of any combination of ligands. In contrast, analysis of the hydrogen-bonding pattern displayed striking effects of ligand binding (Figures 5 and S6). The native state shows a structure with strong stability: most of the structural elements are built from hydrogen bonds of 4.1 kcal/mol or greater. The stability of the structure is slightly enhanced when mannose is bound (0.3 kcal/mol), or when one monomer binds to ATP (overall increase of 0.2 kcal/mol). In contrast, when the mannose-free protein binds a second molecule of ATP, there is a loss of stability (−0.5 kcal/mol compared with one ATP bound; Figure 5C). In the case of the mannose-bound WcbL, binding of a single ATP molecule results in a large loss of stability, equivalent to having two ATP molecules bound (Figure 5D). No further loss of stability is seen with binding of a second ATP.

Drug-like Fragments Can Bind to and Inhibit WcbL

As a first step toward producing novel interventions against WcbL and its homologs, we attempted to discover fragments that could inhibit the enzyme. Assays were undertaken using our activity assay, and DSF screening of WcbL. Analysis in duplicate of more than 850 fragments showed that 29 fragments reproducibly appeared as hits in both assays (Figure 6A). Following an initial rescreening of 16 representative compounds (data not shown), we selected the best inhibitor, 3-cyanochromone, for further study. Four similar compounds are commercially available. Testing of these showed that one neighbor, 6-bromo-3-cyanochromone, has a similar activity, while the others (chromone, chromone-3-carboxaldehyde, and 6,8-dichloro-3-cyanochromone) show significantly weaker activity against WcbL (Figure 6B). We determined that the IC_{50} values for the two most potent inhibitors, 3-cyanochromone and 6-bromo-3-cyanochromone, were 28 ± 1.2 μM and 35 ± 1.1 μM, respectively (Figure 6C), while in a competitive enzyme-inhibition model these showed inhibition constants K_i of 10 ± 6 μM and 12 ± 5 μM (Figure 6D). These compounds were delivering low micromolar affinities for the protein, despite their low size, indicating that these are promising scaffolds for new pharmaceuticals. The ligand efficiency of 3-cyanochromone, at more than 0.4 kJ/mol/atom, is very promising, and suggests that these compounds have a good potential for further development.

DISCUSSION

The *B. pseudomallei* CPS consists of a linear repeat of -3)-2-*O*-acetyl-6-deoxy-β-D-*manno*-heptopyranose-(1-. The genes required for the biosynthesis of this polysaccharide have been mapped in *B. pseudomallei* (Reckseidler et al., 2001) and *Burkholderia mallei* (DeShazer et al., 2001). *wcbL* is one of the genes involved in the synthesis of the precursor sugar GDP-6-deoxy-α-D-*manno*-heptopyranose (dDHep) from S7P, a pentose phosphate pathway intermediate (Cuccui et al., 2012). Analysis of the homologous operon from *Y. pseudotuberculosis* (Pacinelli et al., 2002) suggested that the WcbL homolog would act as a sugar kinase, adding a phosphate to the 1-*O* position of D-α,β-D-heptose-7-phosphate. This has been proved catalytically, as the complete enzymatic synthesis of GDP-6-deoxy-α-D-heptopyranose

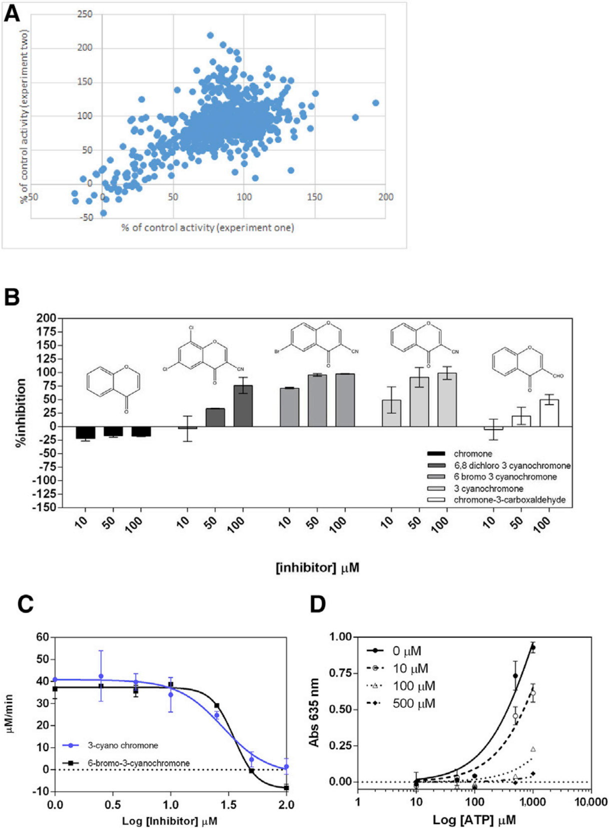

Figure 6. Small Molecules Can Inhibit WcbL

(A) Analysis in duplicate of over 850 fragments from the Zenobia and Maybridge fragment libraries showed reasonable reproducibility. 29 fragments showed reproducible reduction of WcbL activity to less than 25% of control.

(B) Structure-activity relationship studies. Chromone derivatives were screened against WcbL. The compounds were tested at final concentrations of 10, 50, and 100 μM. The percentage of inhibition was calculated from the equation $(1 - V_i/V_0) \times 100$, where V_i and V_0 are the enzyme rates in the presence and absence of inhibitors, respectively. The percentage of inhibition was determined in triplicate.

(C) Dose-response curves for 3-cyanochromone (blue) and 6-bromo-3-cyanochromone (black) against WcbL. Assays were performed in triplicate using the compounds in the concentration range 0–100 μM.

(D) Kinetic assay plots for the inhibitor 3-cyanochromone. The absorbance at 635 nm was plotted as a function of Log_{10}[ATP]. The assay was performed in triplicate using the following concentrations: 0 (filled circle), 10 (empty circle), 100 (empty triangle), or 500 μM (filled rhombus). The fitted data suggested a competitive inhibition with an apparent inhibition constant K_i of 10 ± 6 μM.

Error bars represent SEM.

from S7P has been reported (Butty et al., 2009). While two structures of other putative enzymes with the same predicted activity as WcbL have been reported (PDB: 3K85 and 4N3O), neither of these structures provided any insight into the binding of substrates to the protein or the catalytic mechanism. WcbL represents an excellent target for novel antimicrobials: seven membered sugars are rarely used in eukaryotes, and humans have no equivalent to this pathway (Durka et al., 2011; Lee et al., 2013; Taylor and Wright, 2008). Furthermore, kinases are among the best validated drug targets, representing approximately one-third of protein targets currently under investigation for drug development (Fabbro et al., 2012), while none of the other enzymes in the pathway belong to classes that have previously had successful drugs discovered. Ligand screening in the equivalent ADP-linked sugar pathway found that the equivalent step to WcbL was the easiest for discovering inhibitory compounds (De Leon et al., 2006). A deeper understanding of the reaction, structure, and interactions of this enzyme would therefore offer a strong boost to attempts to develop novel antimicrobials targeting this pathway.

The crystal structure of WcbL from *B. pseudomallei* reported here shows that the enzyme indeed has the expected GHMP sugar kinase fold (Figures 2A and 2B). The protein strongly resembles the two orthologs solved by structural genomics consortia. This gives us strong confidence that the structure and conformation that we have identified represents the native structure of this enzyme.

We then solved the structure of WcbL co-crystallized with the substrate analog AMP-PNP and soaked with D-mannose (Figure 3). These two analogs bind to the enzyme in the locations expected by analogy to previously determined complexes of sugar kinases and their substrates. Notably, WcbL appears to bind to the α-anomer of M7P. This anomer presents the 1-*O* atom of M7P in a suitable conformation for reaction. This fits well with our previously determined structure of GmhA, the previous enzyme in the pathway (Harmer, 2010). In this structure, GmhA appears to specifically catalyze the formation of the α-anomer of M7P, supporting the conjecture that *B. pseudomallei* GmhA catalyzes the closure of the sugar ring as well as isomerization from the keto-to aldo sugar.

We then analyzed the interactions of WcbL and reactions of WcbL. Analysis of reaction kinetics showed that WcbL is cooperative with respect to both M7P and ATP, with *h* values of 1.5 and 1.9, respectively (Figures 4B and 4C). As the predicted ternary complex of WcbL, M7P, and ATP suggests that the binding of ATP blocks access to the binding site for M7P, we analyzed the manner of the reaction. Using the product ADP to determine the effects of product inhibition, we demonstrated that this acts as an uncompetitive/non-competitive inhibitor (Figure S5). Were the reaction a random Bi-Bi reaction, or a ping-pong reaction whereby M7P binds last, this would be expected to be a competitive inhibitor (Cook and Cleland, 2007). This therefore strongly suggests that the reaction is indeed an ordered sequential Bi-Bi reaction, where M7P binds first, then ATP. Intriguingly, our biophysical analysis of the binding of ATP to WcbL showed that ATP binding to WcbL is perfectly anti-competitive (Figure 4A). This suggests that the interactions of WcbL with its substrates are coordinated: in the absence of M7P, binding of ATP is discouraged, as this blocks the M7P binding site, while in the presence of the sugar-phosphate the ATP binding and reaction are strongly cooperative.

We then used modeling approaches to further understand the physical basis for these unusual cooperativity effects. We examined normal modes and hydrogen-bonding networks, as these have previously explained functional effects in a number of systems (e.g. Wells et al., 2014). Surprisingly, the binding of ligands to WcbL did not affect the normal modes preferred by the enzyme. The most favored normal modes display considerable movements in the enzyme, some of which involve the closing of the active-site cleft between the two domains, and so are likely

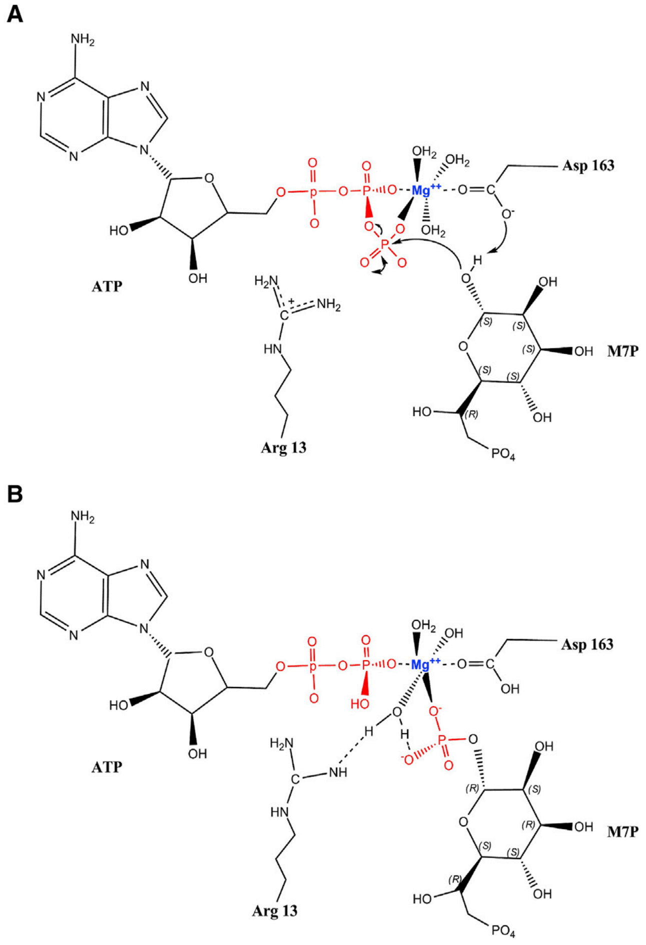

Figure 7. Proposed Mechanism of WcbL Activity

(A) D163 acts as a catalytic base, removing the proton from the M7P 1-*O*. The 1-*O* then attacks the ATP γ-phosphate, forming a pentahedral intermediate that is stabilized by magnesium, and the side chains of R13 and D163.

(B) Finally, this resolves to form the products, with D163 stabilizing the now released β-phosphate of ADP.

to be associated with catalysis. The requirement for flexibility for catalysis perhaps explains why these features of the enzyme are not affected by ligand binding. In contrast, analysis of the hydrogen-bonding network showed considerable effects, localized to the ligand binding regions. Strikingly, the binding of one ATP in the absence of the sugar substrate leads to a tightening of the hydrogen-bonding network and increased the stability of the protomer binding ATP (Figure S6). Binding of a second ATP, in contrast, requires a considerable loss of stability in the protein (Figures 5B and 5C). This effect may explain the observation that ATP binding is strongly anti-cooperative in the absence of the sugar substrate. Binding of the sugar causes a strong stabilization effect. This stabilization is lost, and the hydrogen-bonding network resembles the two-ATP bound state, when a single ATP molecule binds. This suggests that, in the presence of mannose, the binding of a first ATP requires the relaxation of the hydrogen-bonding network, and no further penalty is required to bind a second ATP. This appears to be consistent with the positive cooperativity observed in the enzyme assays (Figures 5B–5D and S6). It therefore appears that the binding of the sugar substrate triggers a switch in the hydrogen-bonding network of the enzyme, so that ATP binding is favored and becomes cooperative, whereas in the absence of sugar, ATP binding at one site disfavors binding at the other site. Both the structure and catalytic properties of WcbL suggest that it obeys an ordered sequential Bi-Bi mechanism whereby the sugar must be loaded into the active site before ATP binding. It is therefore essential for enzyme activity that the binding of ATP to both sites is disfavored in the absence of the sugar substrate, while it will be favorable in the presence of the sugar. The observed $K_{1/2}$ values for both ATP binding in the absence of sugar and for ATP concentration during catalysis are in the mid-micromolar range and well below the usual cellular concentration of ATP. This elegant mechanism of switching the cooperative behavior on binding of the sugar substrate therefore provides the enzyme with ATP interaction behavior appropriate for its mechanism and cellular context.

We attempted to validate the complex and mechanism suggested by the crystal structure. We made mutants of several proposed critical binding and active-site residues for both ATP and M7P. All of these mutants purified well, but showed a complete loss of activity in the enzymatic assay (at least 150-fold below the wild-type; Figure 4D). To further validate this, we investigated the capacity of these mutants to complement a knockout of WcbL in *B. pseudomallei*. *wcbl* mutants of *B. pseudomallei* are unable to produce CPS (Cuccui et al., 2007). Complementation with wild-type WcbL showed a restoration of the capsule production phenotype (Figures 4E and 4F). However, complementation with WcbL with any of these mutations failed to restore any CPS production.

Based on these observations, we propose a mechanism for the activity of WcbL (Figure 7). We propose that D163 acts as a catalytic base, removing the proton from the M7P 1-*O*. The 1-*O* then attacks the ATP γ-phosphate, forming a pentahedral intermediate that is stabilized by magnesium and the side chains of R13 and D163. Finally, this resolves to form the products, with D163 stabilizing the now released β-phosphate of ADP. This model is consistent with all of the structural and kinetic data. Similar mechanisms have been observed with other sugar kinases (Huang et al., 2013; Megarity et al., 2011).

Finally, we used our knowledge of the reactions and structure of WcbL to search for potential inhibitors. Kinases have been shown to be excellent targets for fragment screening, as they often provide very avid binders. A screen of more than 850 compounds by DSF and enzyme assays highlighted a range of potential inhibitors that showed activity at millimolar concentrations. Further testing of these showed that one, 3-cyanochromone, inhibited WcbL at low micromolar concentrations (Figures 6C and 6D). Testing of commercially available analogs of this compound showed that the cyano group is highly important to the interaction, as chromone-3-carboxaldehyde showed a much weaker IC_{50} than the cyano equivalent, while chromone itself does not inhibit WcbL. The 8-bromo equivalent of 3-cyanochromone showed IC_{50} and K_i a very similar to those of the original compound, indicating that the 8-position is suitable for modification, while the 6,8-dichloro equivalent is a far less potent inhibitor, indicating that the 6-position of the chromone ring is not suitable for modification (Figure 6B). These observations

indicate that there is a good potential for developing specific inhibitors for WcbL: a very moderate screen has identified a molecule with good potency, and a very high ligand efficiency of more than 0.4 kJ/mol/heavy atom. With a more extensive screening, it is likely that a very potent inhibitor of WcbL could be identified. This would be a very attractive proposition, offering the potential to deliver a compound that could render pathogenic microorganisms non-infective with minimal damage to commensal organisms.

Our study reveals the elegant regulation of ATP binding that WcbL uses to tune its ligand occupation. The exquisite arrangement of the hydrogen-bonding network allows WcbL to operate a sequentially ordered Bi-Bi kinetic mechanism, with positive cooperativity with respect to both substrates. The structure of WcbL reveals a deep binding pocket for the substrate M7P, with specific changes to the structure to accommodate a heptose rather than a hexose sugar. Based on the structural and kinetic data presented herein, we have proposed a mechanism for the action of WcbL, and show that small drug-like fragments are competent in binding to the active site and inhibiting WcbL in competition with ATP. Given that WcbL knockouts present a very strong phenotype, these data strongly suggest that WcbL would be an excellent target for adjunct therapies to prevent the formation of protective surface polysaccharides in Gram-negative bacteria.

SIGNIFICANCE

This study reports the structure and functional characterization of WcbL. This enzyme is critical in capsular polysaccharide biosynthesis in the human pathogen *Burkholderia pseudomallei*. Our most significant finding is that this enzyme regulates substrate binding order through the global protein structure. This concept has potential implications for bisubstrate enzymes: these constitute around 60% of all enzymes, and most of these require a defined binding order. We further elucidate a likely enzyme activity mechanism, validated both in vitro and in vivo. Finally, we demonstrate that small molecules can inhibit WcbL with high affinity, and show at least one scaffold that can act as a potential lead compound. Our observations validate this enzyme as an excellent target for antimicrobial development.

EXPERIMENTAL PROCEDURES

Construct Cloning

Full-length *wcbL* from *B. pseudomallei* strain K96423 (gift of Rick Titball, University of Exeter) was cloned into the pNIC28-Bsa4 expression vector using ligation-independent cloning (Savitsky et al., 2010).

The *wcbL*-pNIC28 construct was used for site-directed mutagenesis using the QuikChange Lightning II site-directed mutagenesis kit (Agilent). Primers (Life Technologies) were designed using the primer design tool available at http://www.genomics.agilent.com/primerDesignProgram.jsp. All mutants were verified by sequencing.

Constructs for expression in *B. pseudomallei* were prepared by PCR amplifying the wild-type and site-directed mutants of *wcbL* with extensions that provided a ribosome binding site for *Burkholderia*, and sequence synonymous with the multiple cloning site of pBBR1-MCS2 (Kovach et al., 1995). The pBBR1-MCS2 plasmid was also amplified by PCR. Both PCR products were treated with *Dpn*I (Fermentas) to eliminate remaining original plasmid. The PCR products were gel purified, and the concentration determined using a Nanodrop ND2000c spectrophotometer. Plasmid and insert were mixed in a 1:3 M ratio, and cloned using the Gibson cloning kit (NEB) using the manufacturer's recommendations. All successful constructs were verified by sequencing.

Expression and Purification

Constructs were transformed into Rosetta (DE3) cells (Merck) and grown in ZYM-5052 media (Studier, 2005) supplemented with 100 μg/ml kanamycin and 20 μg/ml chloramphenicol. Cells were grown at 37°C until OD_{600} was 0.6 and then at 20°C. Bacteria were harvested after 20 hr, and suspended in 20 mM Tris-HCl (pH 8.0) and 0.5 M NaCl (buffer A). The cells were lysed using a Soniprep 150 sonicator (MSE). The lysate was clarified by centrifuging at 20,000 × *g* for 30 min. The supernatant was loaded over a HisTrap Crude FF column (1 ml) on an ÄKTAxpress chromatography system (GE Healthcare), or to nickel-Sepharose beads (1 ml; GE Healthcare) in a gravity flow column. The column was washed with buffer A supplemented with 25 mM imidazole-HCl (pH 8.0), and the protein eluted with buffer A supplemented with 250 mM imidazole-HCl (pH 8.0). The peak was loaded onto a Superdex 200 16/60-hr size-exclusion column (GE Healthcare) and eluted isocratically into 10 mM HEPES (pH 7.0), 0.5 M NaCl, and 5 mM citrate (pH 6.5). An E179A/E180A/E181A triple mutant protein was purified as above but without addition of citrate at the size-exclusion step. Selenomethionine-labeled WcbL was prepared using PASM-5052 media (Studier, 2005), and purified as above. GmhA and WcbN were prepared as described by Harmer (2010).

Crystallization and Structure Determination

WcbL proved to crystallize spontaneously at concentrations exceeding 5 mg/ml at 4°C in the buffer used for size-exclusion chromatography. Crystallization was controlled by concentrating samples freshly each day from a stock at approximately 1 mg/ml, and by incubating drops of 1–4 μl under oil in microbatch plates (Douglas Instruments). The E179A/E180A/E181A triple mutant crystallized spontaneously, and in a more reproducible manner, at concentrations of around 3 mg/ml. Soaking was attempted by adding substances dissolved in cryoprotectant (see Table S1) to protein at concentrations of 1–25 mM, and incubating at 4°C for up to 90 min. Co-crystallization was performed by adding compounds to WcbL E179A/E180A/E181A pre-concentrated to approximately 3 mg/ml, and adding partner molecules to the concentrations required. Co-crystals formed naturally at 4°C under oil. Either glycerol or mixture 5 from the CryoProtX kit (Molecular Dimensions) was used as a cryoprotectant. Soaking with mannose was achieved by utilizing 30% (w/v) D-mannose as a cryoprotectant.

Data were collected on beamlines I03 and I04-1 at the Diamond Synchrotron light source (Didcot, UK) at 100 K in a stream of gaseous nitrogen using ADSC (for glycerol complex and selenomethionine data) or Pilatus detectors. Data were processed and scaled using XDS (Kabsch, 2010) and AIMLESS (Evans and Murshudov, 2013) in the Xia2 (Winter et al., 2013) pipeline. Further data and model manipulation was carried out using PHENIX (Adams et al., 2010) and the CCP4 suite of programs (Winn et al., 2011).

While several wavelengths were collected from a single crystal of selenomethionine protein, the WcbL structure was solved by SAD in the SHELXC/D/E pipeline (Sheldrick, 2010) implemented in HKL2MAP (Pape and Schneider, 2004) using the selenomethionine high-energy remote data and glycerol complex as native. For the correct hand, the correlation for the partial structure against the native data was 54.45%. The full atomic model was built using ARP/wARP (Langer et al., 2013; Murshudov et al., 2011). Poorly defined loops were rebuilt with the help of BUSTER (Bricogne et al., 2011). Model rebuilding was done in Coot (Emsley et al., 2010). The dictionary definitions for the ligands were created using JLIGAND (Lebedev et al., 2012).

Differential Scanning Fluorimetry

DFS was performed using a StepOne qPCR machine (Applied Biosystems). In brief, purified WcbL was diluted to 0.1 mg/ml in 10 mM HEPES (pH 7.0), 150 mM NaCl, and 8× SYPRO Orange dye (Invitrogen; Niesen et al., 2007), in a final volume of 20 μl. ATP was added to a final concentration between 0.1 μM and 5 mM. All samples were prepared in triplicate. To generate the melting curve, the samples where heated in a gradient from 25° to 99°C over 50 min and the fluorescence measured every 0.4°C. The melting temperature was determined from the peaks of the first derivatives of the melting

curve using Protein Thermal Shift software version 1.0 (Applied Biosystems), and plots were produced using the program GraphPad Prism 6.0.2. The dissociation constant K_D was calculated according to the equation

$$T_m = \text{Bottom} + \left((\text{Top} - \text{Bottom}) * \frac{\left(\frac{[\text{ligand}]}{K_D}\right)^n}{1 + \left(\frac{[\text{ligand}]}{K_D}\right)^n} \right), \qquad \text{(Equation 1)}$$

where K_D is the dissociation constant, in the same units that were used for the ligand concentrations; Top and Bottom are the melting temperatures at infinite ligand concentration and no ligand concentration, respectively, and n represents the Hill coefficient describing cooperativity (Vivoli et al., 2014b).

Kinetic Assays

WcbL activity was measured using a discontinuous coupled system with WcbN, using ATP and D-*manno*-α,β-D-heptose-7-phosphate (M7P) as substrates. The activity was measured with the Pi Colorlock Gold colorimetric assay kit (Innova Biosciences). Reactions were performed in 100 μl, and consisted of 10 mM HEPES (pH 8.0), 10 mM $CaCl_2$, 10 mM $MgCl_2$, 6 μg/ml WcbL, 80 μg/ml WcbN, 1 mM ATP and 0–500 μM M7P, or 250 μM M7P and 0–1 mM ATP. WcbL mutants were used at a final concentration of 60 μg/ml. Assays were performed in triplicate, at 37°C for 10 min, followed by incubation for 10 min at 70°C to stop the reaction. Pi Colorlock Gold mix (17 μl) was added to the assay mixture (68 μl) and incubated for 5 min on ice. Stabilization buffer (6.8 μl) was added, the reaction mix incubated for 10 min at room temperature, and the absorbance at 635 nm measured using a Tecan Infinite M200 microplate reader. Rates were calculated using a standard curve of phosphate. Reactions in which ATP, M7P, or recombinant WcbL were omitted served as controls. The data were fitted using Michaelis-Menten and cooperative sigmoidal equations to extrapolate $K_{1/2}$ and V_{max} using GraphPad Prism version 6.0.2.

Medium Throughput Screening for WcbL Inhibitors

Compound Sources

Compounds were taken from the Zenobia fragment library 1 (Zenobia Fragment Libraries, 352 compounds), and from the Maybridge Ro3 fragment library 1 (Thermo Fisher, 500 compounds). The Zenobia library was prepared in DMSO, and a working stock at 50 mM prepared; the Maybridge library was prepared in DMSO, and a working stock at 25 mM prepared (both according to the manufacturer's recommendations).

Enzymatic Assay

An enzymatic assay of WcbL was performed in a manner similar to that described above. GmhA was used to convert the commercially available substrate sedoheptulose-7-phosphate (S7P) into M7P. Reactions were performed in 100 μl, and consisted of 10 mM HEPES (pH 8.0), 10 mM $CaCl_2$, 10 mM $MgCl_2$, 52.5 μg/ml GmhA, 6 μg/ml WcbL, 34 μg/ml WcbN, 200 μM ATP, and 500 μM S7P. Inhibitors were added to a total of 4% of the reaction volume (2 mM for Zenobia library, 1 mM for Maybridge library). Controls were prepared with 4% DMSO. Assays were performed in 96-well plates, with each plate containing four positive control wells and four negative control plates (lacking WcbL). Reactions were performed as described above, and analyzed using the Pi Colorlock Gold detection reagent. All experiments were performed in duplicate, and compounds considered as hits if they reduced the rate of reaction to less than 25% in both replicates.

DSF Binding Assay

DSF assays were set up in 96-well white qPCR plates (Fisher) and sealed with optical tape (Bio-Rad). Samples contained 1 μM WcbL, 10 mM HEPES (pH 7.0), 150 mM NaCl, and 5× SYPRO Orange in a final reaction volume of 40 μl. Compounds were added to 10% of the working concentration (2.5 mM for Maybridge library and 5 mM for Zenobia library). The DSF reactions were performed using an Mx3000 qPCR machine (Agilent; experimental parameters: 25°C for 5 min, then 1°C rise per minute to 95°C). Data were analyzed using the methods of Niesen et al. (2007). T_m values were calculated by non-linear regression, fitting the Boltzmann equation to the melting curves using GraphPad Prism version 5. All experiments were performed in duplicate. Compounds were considered as hits if both samples gave melting temperatures raised above 42°C (88th percentile of all samples tested; WcbL treated with DMSO alone gave a melting temperature of 37°C ± 1°C).

Compound Selection, Structure-Activity Relationship Studies, and Determination of IC_{50} and K_i Values for the Best Inhibitors

General Assay Conditions

Compounds at appropriate concentrations (see below) were placed into plates containing WcbL (6 μg/ml), GmhA (60 μg/ml) and WcbN (80 μg/ml). After a 30-min preincubation at room temperature, S7P (final concentration 500 μM) and ATP (final concentration 1 mM) were added, and residual enzyme activity was monitored using the Pi Colorlock reagent as described above.

Compound Selection

Compounds that were considered "hits" in both of the initial screens were selected for more detailed studies. Fresh samples of each compound were obtained, and the compounds retested using concentrations of compound between 20 μM and 5 mM.

Structure-Activity Relationship Study

Compounds were added at concentrations of 10, 50, and 100 μM. The percentage of inhibition was calculated from the equation $(1 - V_i/V_o) \times 100$, where V_i and V_o are the enzyme rates in the presence and in the absence of inhibitors, respectively. Each point was determined in triplicate.

Dose-Response Experiments

Compounds 3-cyanochromone and 6-bromo-3-cyanochromone were added at serial dilutions to give final concentrations between 1 and 100 μM. The data obtained were fitted using GraphPad Prism version 6.0.2, applying the following four-parameter logistic equation for a standard dose-response curve:

$$\text{Response} = \text{Bottom} + \frac{(\text{Top} - \text{Bottom})}{1 + 10^{(\log_{10}\text{IC}_{50} - \log_{10}[I]) * h}}, \qquad \text{(Equation 2)}$$

where the Response decreases as [I] (inhibitor concentration) increases; Top and Bottom are the plateaus in same units as Response; $\text{Log}_{10}\text{IC}_{50}$ is in the same log units as [I]; and h is the slope factor, unitless.

K_i determination

To determine the inhibition constant for 3-cyanochromone, compound was used at 0, 10, 100, and 500 μM as described above. The ATP concentration used in the reaction was varied from 0 to 1 mM, and enzyme activity was detected as described above. The K_i value was calculated using the equation for competitive inhibition:

$$v = V_{max} * \frac{[S]}{\left(\left(K_M * \left(1 + \frac{[I]}{K_i}\right)\right) + [S]\right)}, \qquad \text{(Equation 3)}$$

where V_{max} is the maximum enzyme velocity; [S] is the substrate concentration; K_M is the Michaelis constant (same units as [I]); [I] is the inhibitor concentration; and K_i is the inhibition constant.

The K_i for 6-bromo-3-cyanochromone was extrapolated using the following equation, assuming a competitive inhibition:

$$K_i = \frac{\text{IC}_{50}}{\left(\frac{S}{K_M + 1}\right)}. \qquad \text{(Equation 4)}$$

Mechanistic Studies

Kinetic studies of the reaction mechanism of WcbL were performed by product inhibition experiments using the assay methodology outlined above. For these experiments, ADP was used as the product inhibitor. In brief, ADP at concentrations of 100, 500, and 1,000 μM was placed into plates containing 12 μg/ml WcbL, 80 μg/ml WcbN, 1 mM ATP and 0–250 μM M7P, or 250 μM M7P and 0–1 mM ATP, and residual enzyme activity monitored using the Pi Colorlock reagent as described above. The initial rates derived from these inhibition experiments were fitted to equations representative of competitive, non-competitive, mixed, or uncompetitive inhibition by a non-linear least-squares approach using GraphPad Prism version 6.0.2. The best fits of the data were compared using a combination of visual inspection, comparison of R^2 value, and Akaike's Information Criteria.

In Vivo Assays

Complementation of the previously described *B. pseudomallei* *wcbL*$^-$ mutant (Cuccui et al., 2007) was completed using tri-parental mating. In brief, pBBR1-MCS2 complementation vectors (see above) were transformed into

Escherichia coli JM109 and selected using 50 μg/ml kanamycin. A 10 μl aliquot from an overnight culture of a given donor strain, the *B. pseudomallei* K96243 Δ*wcbL* recipient strain, and the *E. coli* HB101 (pRK2013) helper strain were inoculated as overlapping spots onto a nitrocellulose membrane previously placed on the surface of an L-agar plate. After an overnight incubation at 37°C, the bacterial growth was harvested into PBS (1 ml) and aliquots spread onto L-agar containing 50 μg/ml gentamicin and 750 μg/ml kanamycin.

The phenotype of the complemented strains was assessed using dot-blot western hybridization. In brief, the complemented *B. pseudomallei* Δ*wcbL* isolates were incubated in L-broth containing 750 μg/ml kanamycin overnight at 37°C. Cells were pelleted, suspended in PBS, and inactivated by heating to 80°C for 4 hr. Once cooled, aliquots of each isolate (10 μl) were dried onto a nitrocellulose membrane and the membranes used for western hybridization as previously described (Nelson et al., 2004), using the mouse-derived capsule-specific monoclonal antibody 4VIH12 and O antigen specific monoclonal antibody CC6 (Ellis, 2000) for detection. SigmaFast DAB (Sigma) was used for development of the membranes.

Computational Studies

Samples were prepared for computational analysis by setting all residues with alternative conformations to the most favored conformation using PHENIX. A structure with one AMP-PNP was prepared by removing the AMP-PNP from molecule B, and altering the ions to those present in the native structure. Structures with mannose and one (in molecule B) or two AMP-PMP molecules were prepared by adding the AMP-PNP molecules and ions from each molecule to the relevant molecule in the mannose complex. Explicit hydrogens were added to the structure, and the structure was optimized using the online version of YASARA (Krieger et al., 2009). Normal mode analysis was undertaken using the elNémo server (http://www.sciences.univ-nantes.fr/elnemo/) (Suhre and Sanejouand, 2004), using standard settings. Hydrogen-bonding network analysis was performed using Proflex (Jacobs et al., 2001). Hydrogen-bond dilution was performed using default settings.

ACCESSION NUMBERS

The coordinates and structure factors for the structures reported in this paper have been submitted to the PDB with accession numbers: PDB: 4USK, native WcbL; PDB: 4UTG, WcbL in complex with AMP-PNP; PDB: 4USM, WcbL in complex with glycerol; PDB: 4UT4, WcbL in complex with D473 mannose.

SUPPLEMENTAL INFORMATION

Supplemental Information includes six figures and one table and can be found with this article online at http://dx.doi.org/10.1016/j.chembiol.2015.10.015.

AUTHOR CONTRIBUTIONS

R.N. and N.H. performed the molecular biology; M.V. performed the enzymatic assays; M.V., M.I., and N.H. undertook the crystallization and crystallography; A.H. and N.H. performed the fragment screening; A.S. and J.P. performed the in vivo assays; P.K. synthesized M7P; M.V., M.I., A.S., P.K., and N.H. wrote the manuscript.

ACKNOWLEDGMENTS

The authors thank Ellen Harmer (University of Exeter) for assistance in cloning the mutants used in this study; Richard Eastmont (University of Exeter) for assistance in determining the correct conditions for purification; the staff at Diamond beamlines I03 and I04-1 for assistance in data collection; and Stephen Wells (University of Bath) and Leslie Kuhn (University of Michigan) for discussions regarding the bioinformatics approaches. This work was funded by grants from the Royal Society (2009/R2) and BBSRC (BB/H019685/1) to N.H.

Received: July 3, 2015
Revised: October 20, 2015
Accepted: October 31, 2015
Published: December 17, 2015

REFERENCES

Adams, P.D., Afonine, P.V., Bunkoczi, G., Chen, V.B., Davis, I.W., Echols, N., Headd, J.J., Hung, L.W., Kapral, G.J., Grosse-Kunstleve, R.W., et al. (2010). PHENIX: a comprehensive Python-based system for macromolecular structure solution. Acta Crystallogr. D Biol. Crystallogr. *66*, 213–221.

Atkins, T., Prior, R., Mack, K., Russell, P., Nelson, M., Prior, J., Ellis, J., Oyston, P.C., Dougan, G., and Titball, R.W. (2002). Characterisation of an acapsular mutant of *Burkholderia pseudomallei* identified by signature tagged mutagenesis. J. Med. Microbiol. *51*, 539–547.

Bricogne, G., Blanc, E., Brandl, M., Flensburg, C., Keller, P., Paciorek, W., Roversi, P., Sharff, A., Smart, O., Vonrhein, C., et al. (2011). BUSTER (Global Phasing Ltd.).

Butler, D. (2012). Viral research faces clampdown. Nature *490*, 456.

Butty, F., Aucoin, M., Morrison, L., Ho, N., Shaw, G., and Creuzenet, C. (2009). Elucidating the formation of 6-deoxyheptose: biochemical characterisation of the GDP-D-glycero-D-manno-heptose C6 dehydratase, DmhA, and its associated C4 reductase, DmhB. Biochemistry *48*, 7764–7775.

Chovancova, E., Pavelka, A., Benes, P., Strnad, O., Brezovsky, J., Kozlikova, B., Gora, A., Sustr, V., Klvana, M., Medek, P., et al. (2012). CAVER 3.0: a tool for the analysis of transport pathways in dynamic protein structures. PLoS Comput. Biol. *8*, e1002708.

Cook, P.F., and Cleland, W.W. (2007). Enzyme Kinetics and Mechanism (Garland Science).

Cuccui, J., Easton, A., Chu, K., Bancroft, G., Oyston, P., Titball, R., and Wren, B. (2007). Development of signature-tagged mutagenesis in *Burkholderia pseudomallei* to identify genes important in survival and pathogenesis. Infect. Immun. *75*, 1186–1195.

Cuccui, J., Milne, T.S., Harmer, N., George, A.J., Harding, S.V., Dean, R.E., Scott, A.E., Sarkar-Tyson, M., Wren, B.W., Titball, R.W., et al. (2012). Characterization of the *Burkholderia pseudomallei* K96243 capsular polysaccharide I coding region. Infect. Immun. *80*, 1209–1221.

De Leon, G.P., Elowe, N.H., Koteva, K.P., Valvano, M.A., and Wright, G.D. (2006). An in vitro screen of bacterial lipopolysaccharide biosynthetic enzymes identifies an inhibitor of ADP-heptose biosynthesis. Chem. Biol. *13*, 437–441.

DeShazer, D., Waag, D.M., Fritz, D.L., and Woods, D.E. (2001). Identification of a *Burkholderia mallei* polysaccharide gene cluster by subtractive hybridization and demonstration that the encoded capsule is an essential virulence determinant. Microb. Pathog. *30*, 253–269.

Durka, M., Tikad, A., Perion, R., Bosco, M., Andaloussi, M., Floquet, S., Malacain, E., Moreau, F., Oxoby, M., Gerusz, V., et al. (2011). Systematic synthesis of inhibitors of the two first enzymes of the bacterial heptose biosynthetic pathway: towards antivirulence molecules targeting lipopolysaccharide biosynthesis. Chemistry *17*, 11305–11313.

Ellis, J. (2000). Antibody Detection of *Burkholderia pseudomallei* and *Burkholderia mallei* (Aston University).

Emsley, P., Lohkamp, B., Scott, W.G., and Cowtan, K. (2010). Features and development of Coot. Acta Crystallogr. D Biol. Crystallogr. *66*, 486–501.

Evans, P.R., and Murshudov, G.N. (2013). How good are my data and what is the resolution? Acta Crystallogr. D Biol. Crystallogr. *69*, 1204–1214.

Fabbro, D., Cowan-Jacob, S.W., Mobitz, H., and Martiny-Baron, G. (2012). Targeting cancer with small-molecular-weight kinase inhibitors. Methods Mol. Biol. *795*, 1–34.

Gilbert, M., Mandrell, R.E., Parker, C.T., Li, J., and Vinogradov, E. (2007). Structural analysis of the capsular polysaccharide from *Campylobacter jejuni* RM1221. Chembiochem *8*, 625–631.

Harmer, N.J. (2010). The structure of sedoheptulose-7-phosphate isomerase from *Burkholderia pseudomallei* reveals a zinc binding site at the heart of the active site. J. Mol. Biol. *400*, 379–392.

Holden, M.T.G., Titball, R.W., Peacock, S.J., Cerdeno-Tarraga, A.M., Atkins, T., Crossman, L.C., Pitt, T., Churcher, C., Mungall, K., Bentley, S.D., et al. (2004). From the cover: genomic plasticity of the causative agent of melioidosis, *Burkholderia pseudomallei*. Proc. Natl. Acad. Sci. USA *101*, 14240–14245.

Huang, M., Li, X., Zou, J.W., and Timson, D.J. (2013). Role of Arg228 in the phosphorylation of galactokinase: the mechanism of GHMP kinases by quantum mechanics/molecular mechanics studies. Biochemistry *52*, 4858–4868.

Jacobs, D.J., Rader, A.J., Kuhn, L.A., and Thorpe, M.F. (2001). Protein flexibility predictions using graph theory. Proteins *44*, 150–165.

Kabsch, W. (2010). Xds. Acta Crystallogr. D Biol. Crystallogr. *66*, 125–132.

Kingsley, R.A., and Baumler, A.J. (2000). Host adaptation and the emergence of infectious disease: the Salmonella paradigm. Mol. Microbiol. *36*, 1006–1014.

Kosma, P. (2008). Occurrence, synthesis and biosynthesis of bacterial heptoses. Curr. Org. Chem. *12*, 1021–1039.

Kovach, M.E., Elzer, P.H., Hill, D.S., Robertson, G.T., Farris, M.A., Roop, R.M., 2nd, and Peterson, K.M. (1995). Four new derivatives of the broad-host-range cloning vector pBBR1MCS, carrying different antibiotic-resistance cassettes. Gene *166*, 175–176.

Krieger, E., Joo, K., Lee, J., Lee, J., Raman, S., Thompson, J., Tyka, M., Baker, D., and Karplus, K. (2009). Improving physical realism, stereochemistry, and side-chain accuracy in homology modeling: four approaches that performed well in CASP8. Proteins *77* (*Suppl 9*), 114–122.

Langer, G.G., Hazledine, S., Wiegels, T., Carolan, C., and Lamzin, V.S. (2013). Visual automated macromolecular model building. Acta Crystallogr. D Biol. Crystallogr. *69*, 635–641.

Lazar Adler, N.R., Govan, B., Cullinane, M., Harper, M., Adler, B., and Boyce, J.D. (2009). The molecular and cellular basis of pathogenesis in melioidosis: how does *Burkholderia pseudomallei* cause disease? FEMS Microbiol. Rev. *33*, 1079–1099.

Lebedev, A.A., Young, P., Isupov, M.N., Moroz, O.V., Vagin, A.A., and Murshudov, G.N. (2012). JLigand: a graphical tool for the CCP4 template-restraint library. Acta Crystallogr. D Biol. Crystallogr. *68*, 431–440.

Lee, T.W., Verhey, T.B., Antiperovitch, P.A., Atamanyuk, D., Desroy, N., Oliveira, C., Denis, A., Gerusz, V., Drocourt, E., Loutet, S.A., et al. (2013). Structural-functional studies of *Burkholderia cenocepacia* D-glycero-beta-D-manno-heptose 7-phosphate kinase (HldA) and characterization of inhibitors with antibiotic adjuvant and antivirulence properties. J. Med. Chem. *56*, 1405–1417.

Limmathurotsakul, D., and Peacock, S.J. (2011). Melioidosis: a clinical overview. Br. Med. Bull. *99*, 125–139.

Lovering, A.L., Safadi, S.S., and Strynadka, N.C. (2012). Structural perspective of peptidoglycan biosynthesis and assembly. Annu. Rev. Biochem. *81*, 451–478.

Matsuura, M. (2013). Structural modifications of bacterial lipopolysaccharide that facilitate gram-negative bacteria evasion of host innate immunity. Front. Immunol. *4*, 109.

Megarity, C.F., Huang, M., Warnock, C., and Timson, D.J. (2011). The role of the active site residues in human galactokinase: implications for the mechanisms of GHMP kinases. Bioorg. Chem. *39*, 120–126.

Murshudov, G.N., Skubak, P., Lebedev, A.A., Pannu, N.S., Steiner, R.A., Nicholls, R.A., Winn, M.D., Long, F., and Vagin, A.A. (2011). REFMAC5 for the refinement of macromolecular crystal structures. Acta Crystallogr. D Biol. Crystallogr. *67*, 355–367.

Nelson, M., Prior, J., Lever, M.S., Jones, H.E., Atkins, T.P., and Titball, R.W. (2004). Evaluation of lipopolysaccharide and capsular polysaccharide as subunit vaccines against experimental melioidosis. J. Med. Microbiol. *53*, 1177–1182.

Niesen, F.H., Bergland, H., and Vedadi, M. (2007). The use of differential scanning fluorimetry to detect ligand interactions that promote protein stability. Nat. Protoc. *2*, 2212–2221.

Pacinelli, E., Wang, L., and Reeves, P.R. (2002). Relationship of *Yersinia pseudotuberculosis* O Antigens IA, IIA, and IVB: the IIA gene cluster was derived from that of IVB. Infect. Immun. *70*, 3271–3276.

Pape, T., and Schneider, T.R. (2004). HKL2MAP: a graphical user interface for phasing with SHELX programs. J. Appl. Crystallogr. *37*, 843–844.

Raetz, C.R., and Whitfield, C. (2002). Lipopolysaccharide endotoxins. Annu. Rev. Biochem. *71*, 635–700.

Reckseidler, S.L., DeShazer, D., Sokol, P.A., and Woods, D.E. (2001). Detection of bacterial virulence genes by subtractive hybridization: identification of capsular polysaccharide of *Burkholderia pseudomallei* as a major virulence determinant. Infect. Immun. *69*, 34–44.

Rotz, L.D., Khan, A.S., Lillibridge, S.R., Ostroff, S.M., and Hughes, J.M. (2002). Public health assessment of potential biological terror agents. Emerg. Infect. Dis. *8*, 225–230.

Savitsky, P., Bray, J., Cooper, C.D., Marsden, B.D., Mahajan, P., Burgess-Brown, N.A., and Gileadi, O. (2010). High-throughput production of human proteins for crystallization: the SGC experience. J. Struct. Biol. *172*, 3–13.

Sheldrick, G.M. (2010). Experimental phasing with SHELXC/D/E: combining chain tracing with density modification. Acta Crystallogr. D Biol. Crystallogr. *66*, 479–485.

Stone, R. (2007). Racing to defuse a bacterial time bomb. Science *317*, 1021–1023.

Studier, F.W. (2005). Protein production by auto-induction in high-density shaking cultures. Protein Expr. Purif. *41*, 207–234.

Suhre, K., and Sanejouand, Y.H. (2004). ElNemo: a normal mode web server for protein movement analysis and the generation of templates for molecular replacement. Nucleic Acids Res. *32*, W610–W614.

Taylor, P.L., and Wright, G.D. (2008). Novel approaches to discovery of antibacterial agents. Anim. Health Res. Rev. *9*, 237–246.

Vivoli, M., Ayres, E., Beaumont, E., Isupov, M.N., and Harmer, N.J. (2014a). Structural insights into WcbI, a novel polysaccharide-biosynthesis enzyme. IUCrJ *1*, 28–38.

Vivoli, M., Novak, H.R., Littlechild, J.A., and Harmer, N.J. (2014b). Determination of protein-ligand interactions using differential scanning fluorimetry. J. Vis. Exp. 51809.

Wells, S.A., Crennell, S.J., and Danson, M.J. (2014). Structures of mesophilic and extremophilic citrate synthases reveal rigidity and flexibility for function. Proteins *82*, 2657–2670.

White, N. (2003). Melioidosis. Lancet *361*, 1715–1722.

Whitfield, C. (2006). Biosynthesis and assembly of capsular polysaccharides in *Escherichia coli*. Annu. Rev. Biochem. *75*, 39–68.

Wiersinga, W.J., Currie, B.J., and Peacock, S.J. (2012). Melioidosis. N. Engl. J. Med. *367*, 1035–1044.

Winn, M.D., Ballard, C.C., Cowtan, K.D., Dodson, E.J., Emsley, P., Evans, P.R., Keegan, R.M., Krissinel, E.B., Leslie, A.G., McCoy, A., et al. (2011). Overview of the CCP4 suite and current developments. Acta Crystallogr. D Biol. Crystallogr. *67*, 235–242.

Winter, G., Lobley, C.M., and Prince, S.M. (2013). Decision making in xia2. Acta Crystallogr. D Biol. Crystallogr. *69*, 1260–1273.

Chemistry & Biology, Volume *22*

Supplemental Information

Unraveling the *B. pseudomallei* Heptokinase

WcbL: From Structure to Drug Discovery

Mirella Vivoli, Michail N. Isupov, Rebecca Nicholas, Andrew Hill, Andrew E. Scott, Paul Kosma, Joann L. Prior, and Nicholas J. Harmer

Supplementary Table 1. Data collection and refinement statistics (Figure 2)

[a] Values in parentheses are given for the outer resolution shell.

[*] $R_{sym}=\Sigma_h\Sigma_J|<I_h>-I_J(h)|/\Sigma_h\Sigma_J I(h)$, where I(h) is the intensity of reflection h. Σ_h is the sum over all reflections and Σ_J is the sum over J measurements of the reflection.

[†] Rcryst =Σ||Fo|-|Fc||/Σ|Fo|

[‡] Target values are given in square brackets.

[§] Wilson B-factor was estimated by SFCHECK (Vaguine, et al., 1999).

[¶]Ramachandran plot analysis was performed by *MolProbity* (Chen, et al., 2010).

[#] Cryo5 consist of 12.5 % di-ethylene glycol + 25 % ethylene glycol + 12.5 % MPD + 12.5 % 1,2-propanediol + 12.5 % glycerol + 12.5 mM NDSB 201 (Molecular Dimensions).

**Dataset**	**Se-Met high energy remote**	**Glycerol complex**	**Native**	**Mannose complex**	**AMP-PNP complex**
Beamline (Diamond)	I03	I03	I04-1	I03	I03
Spacegroup	$C222_1$	$C222_1$	$C222_1$	$C222_1$	$C222_1$
Cell parameters (Å)	*a*=101.6, *b*=116.2, *c*=167.7	*a*=101.1, *b*=115.9, *c*=167.4	*a*=101.6, *b*=116.1, *c*=168.2	*a*=101.1 *b*=116.3, *c*=168.1	*a*=101.4 *b*=115.0, *c*=166.8
Protein sequence modification	Se-Met	-	E179A, E180A, E181A	E179A, E180A, E181A	E179A, E180A, E181A
Rotation interval collected °.	360	180	200	180	180
Wavelength (Å)	0.9778	0.9763	0.9200	0.9763	0.9763
Resolution range (Å)	56.5-2.27 (2.33-2.27)	76.2-1.82 (1.87-1.82)	48.6-1.76 (1.81-1.76)	69.5-1.94 (1.99-1.94)	76.1- 1.93 (1.98-1.93)
Completeness (%)	99.7 (99.7)	99.1 (93.8)[a]	98.4 (96.6)	99.6 (99.1)	99.6 (96.7)
Redundancy	14.6 (14.2)	7.2 (5.4)[a]	7.4 (7.6)	6.5 (6.4)	6.5 (6.3)
(I)/σ (I)	15.8 (3.7)	15.4 (1.9)[a]	12.6 (1.9) [a]	7.5 (2.2)	12.8 (2.1)
[*] R_{sym} (%)	0.127 (0.779)	0.078 (0.803) [a]	0.084 (0.915) [a]	0.122 (0.625)[a]	0.082 (0.792)
[†] R_{cryst} (%)		19.2	19.9	19.6	19.6
R_{free} (5% total data; %)		22.9	23.7	23.7	24.2
RMSD bond length[‡] (Å)		0.010 (0.019)	0.012 (0.019)	0.008 (0.019)	0.009 (0.019)
RMSD bond angles[‡] (°)		1.34 (1.96)	1.46 (1.96)	1.22 (1.96)	1.32 (1.96)
Wilson B factor[§] (Å^2)	40.2	30.6	31.3	34.3	36.0
Average B factor (Å^2)					
Protein		24.6	25.0	27.9	30.9
Solvent		39.1	39.5	42.3	44.1
Ligand		34.0	-	32.2	60

Occupancy of ligand		1.0	-	1.0	0.8
Ramachandran plot analysis (%)					
Residues in most favoured regions		98.3	97.8	98.4	98.1
Residues in outlier regions		0.26	0.13	0.13	0.13
MolProbity score [¶] [percentile]		1.75 [82nd]	1.68 [85th]	1.61 [92nd]	1.61 [92nd]
Cryoprotectant (30%)	Cryo5[#]	glycerol	Cryo5[#]	D-mannose	Cryo5[#]

Supplementary Figure 1: Size exclusion chromatography of WcbL (Figure 2)

The behavior of WcbL in size exclusion chromatography was assessed using a Superdex 200 100/30 hr column (GE Healthcare), attached to an ÄKTApurifier instrument (GE Healthcare). WcbL and standard samples were added in a volume of 100 μL, in triplicate. Elution volumes were taken as the point of highest absorbance. The standard curve was fitted to the equation:

$$Log_{10}RMM = (m * \text{elution vol.}) + C$$

Values for the constants m and C were determined from the data by linear regression using GraphPad Prism version 6.0.2. The standards used were dextran (2 MDa), thyroglobulin (667 kDa), apoferritin (443 kDa), alcohol dehydrogenase (150 kDa), albumin (66 kDa), and carbonic anhydrase (29 kDa). Error bars on the figure show standard error. WcbL eluted at a volume consistent with a molecular weight of 117 ± 4 kDa. As the molecular weight of each WcbL protomer is 40.53 kDa, this apparent molecular weight is most consistent with a dimer. The predicted molecular weight is less than would be expected for three protomers forming a highly spherical complex, and the crystal structure shows a dimer that deviates significantly from a sphere. Consequently, a higher hydrodynamic radius would be expected, consistent with the greater apparent molecular weight observed. Error bars represent standard error of the mean.

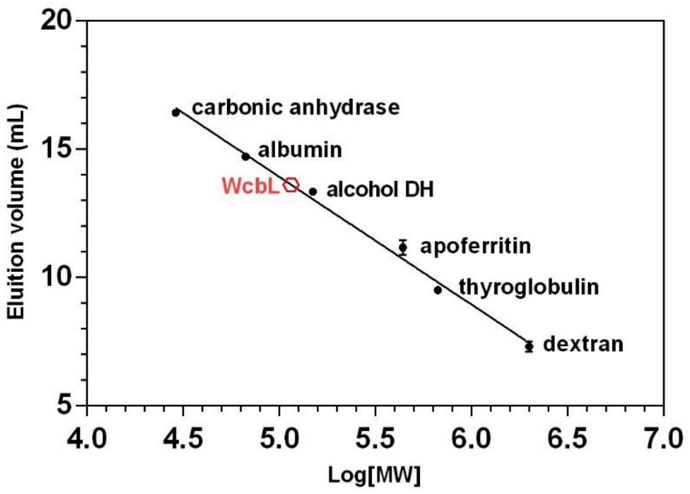

Supplementary Figure 2: Alignment of WcbL with related enzymes of known structure (Figure 3).

The three proteins with the best structural similarity to WcbL were identified using the I-TASSER server (http://zhanglab.ccmb.med.umich.edu/I-TASSER/; Yang, et al., 2015). Two of these (PDB IDs 4N3O and 3K85) are predicted to be kinases of phospho-heptoses from *Campylobacter jejuni* and *Bacteroides thetaiotaomicron* respectively. The third (PDB ID 2DEI) is a galactokinase from *Pyrococcus horikoshii*. An alignment of these three proteins was made guided by the I-TASSER alignments and edited by hand. Residues conserved between all four proteins are highlighted with a cyan background. The residues examined in this study are colored red, and highlighted with a black arrowhead. All of these are generally conserved (all orthologues have two serines in the ATP binding loop) between the four proteins with the exception of Q162 (WcbL), which is conserved in the phosphoheptose kinases, but not in the galactokinase. This is indicated with a red arrowhead.

```
                       ▼        ▼
2CZ9      -----MIKVKSPGRVNLIGEHTDYTY------GYVMPMAINLYTKIEAEK--HGEVILYS
4N3O      SNAMKTIRTQTPLRLGLAGGGTDINLYCDKYTGYVLNATISLYIHCTLIKREDGKIIFDS
WcbL      -MNPTIIRARAPLRLGLAGGGTDVAPYADTFGGYVLNATIDRYAYAVIKTLTIPAVRFVS
3K85      ---MSLVRSKAPLRLGLAGGGSDVSPYSDIYGGLILNATINLYAYCTIEETNSGRIEINA

                                                                 ▼
2CZ9      EHFGEERKFSLNDLRKENSWIDYVKGIFWVLKESDYE---VGGIKGRVSGNLPLGAGLSS
4N3O      PDTNSYCEYESKEFLGNDGKLDIFKSIYNRIV-KDFT-KKPLSFSLHTYSDVPSGSGLGG
WcbL      TDQQVEKHQLISEPLELNGTLNLHKAVYNHMI-RNYNHGKPIALELSTFCDAPAGSGLGS
3K85      YDAQCCKSYLSMSQLEIDGEASLIKGVYNRII-RDYRL-EPKSFKITTYNDAPAGSGLGT

          ▼▼                                         ▼▼
2CZ9      SASFEVGILETLDKLYNLKLDSLSKVLLAKKAENEFVGVPCGILDQFAVVFGREGNVIFL
4N3O      SSTLVVGVIKAFAEWLNLPLGEYEIAKLAYEIEREDLGIVGGAQDQYAATFGGFNFMEFY
WcbL      SSTLVVVMIKAFVELLNLPLDDYAIAQLAYRIERVDCGLAGGRQDQYSATFGGFNFMEFY
3K85      SSTMVVCILKAFIEWLSLPLGDYETSRLAYEIERKDLGLSGGKQDQYAAAFGGFNYMEFL

2CZ9      DTHTLDYEYIPFPK------DVSILVFYTGVRRELASSEYAERKHIAEESLKILGKGSSK
4N3O      NNKRVIVNPLRIKNWIASELEARTVLYFTNITREAKDIEEHKKG----------------
WcbL      EEERTIVNPLRIKNWVLCELEASLVLFYTGVSRESAKIIQDQSDNV--------------
3K85      QNDLVIVNPLKMKRWIVDELESSMVLYFTGRSRSSAAIINEQKKNT--------------

2CZ9      EVREGELSKLPPLHRKFFGYIVRENARVLEVRDALKEGNVEEVGKILTTAHWDLAKNYE-
4N3O      --KLGDEKSLEAMHAI---------KQDAIKMKEALFRADFGTLAQILG-KSWRSKKIISE
WcbL      --VSHKTAAIEAMHGI---------KREALVMKEALLKGDFKAFVASMR-LGWDNKKNSAR
3K85      --SEGNQTAIEAMHKI---------KQSAIDTKLALLKGDVGEFARILG-EGWENKKKMAG

2CZ9      -VSCKELDFFVERALKLGAYGARLTGAGFGGSAIALVDKEDAETIGEEILRE--YLKRFP
4N3O      IVSNDELERIYKLAIDNGAYSGKTSGAGAGGFMFFFVDPTKKYNLIKALRKEQGYVQDF-
WcbL      TVSNAHIDEIYDAAIRAGAQAGKVSGAGGGGFMLFFVPTEKRMDLIRTLGEYDGQVSNC-
3K85      AITNPMIQEAFDVATGAGAMAGKVSGAGGGGFIMFVVEPTRKEEVVRALNNLNGFVMPF-

2CZ9      WKARHFIVEPSDGVGI----------
4N3O      ----SFTKEGVKSWRI----------
WcbL      ----HFTKNGTQAWRIAN--------
3K85      ----QFIDDGAHGWKIYSTDKVQKEG
```

Supplementary Figure 3: Coordination of magnesium (Figure 3). The magnesium ion (green sphere) is shown with neighboring amino acids and AMP-PNP shown as sticks. The protein backbone is shown as cartoon, in the background. The seven atoms at reasonable distances and orientations for coordination interactions are shown as dashed lines, with the distance in Å indicated. Colors: red, oxygen; blue, nitrogen; orange, phosphorus; white, protein carbon; yellow, AMP-PNP carbon. Image produced using PyMOL.

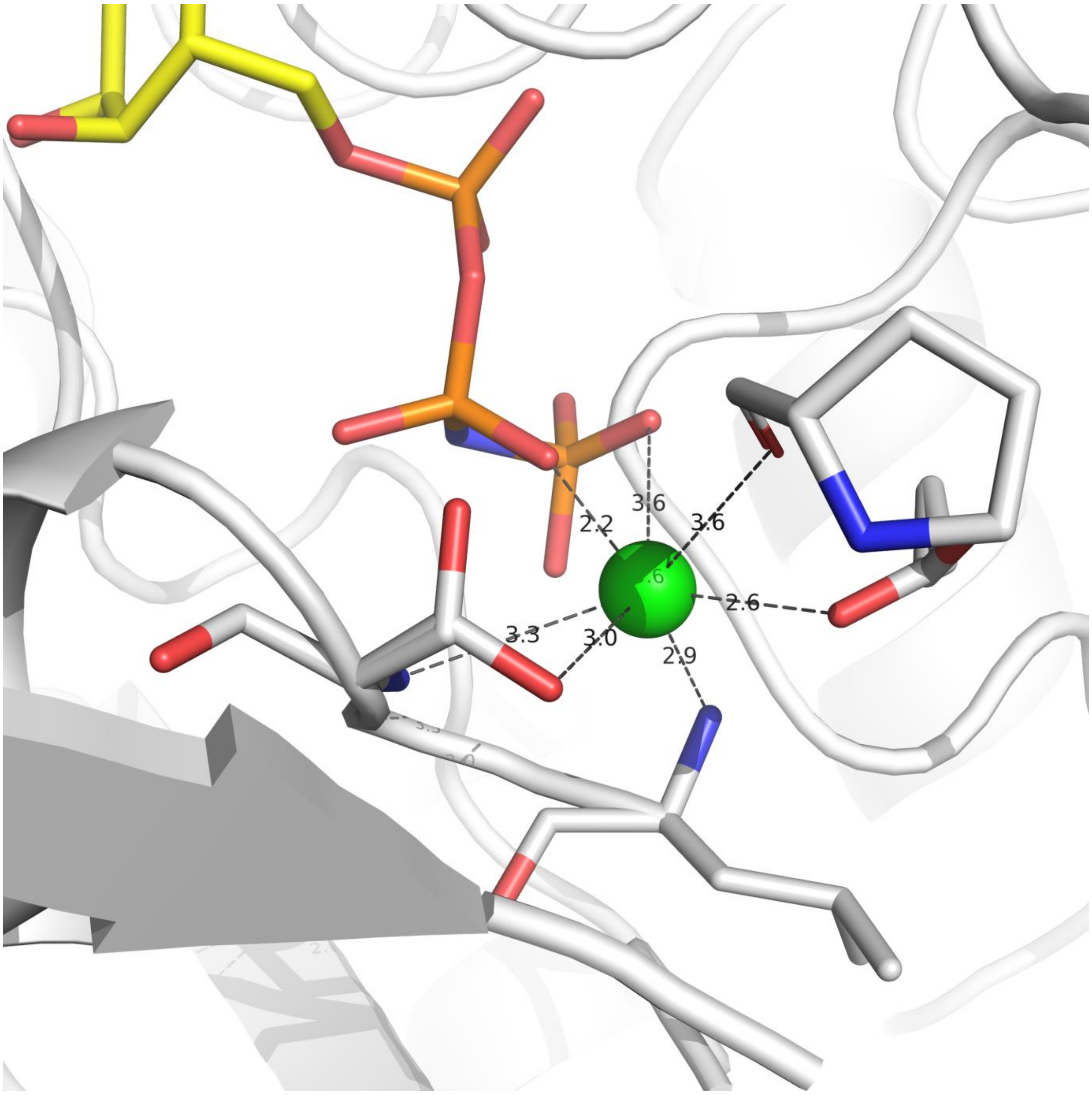

Supplementary Figure 4: Soaked glycerol occupies similar locations to soaked mannose (Figure 3). The structures of complexes with mannose (yellow carbon) and glycerol (green carbon) are shown superimposed. Protein side chains forming hydrogen bonds with either ligand are shown as sticks, and hydrogen bonds with either ligand are indicated by dashed lines, with the distance between heavy atoms in Å indicated. Colors: red, oxygen; blue, nitrogen; orange, phosphorus; purple, mannose soak protein carbon; cyan, glycerol soak protein carbon. Image produced using PyMOL.

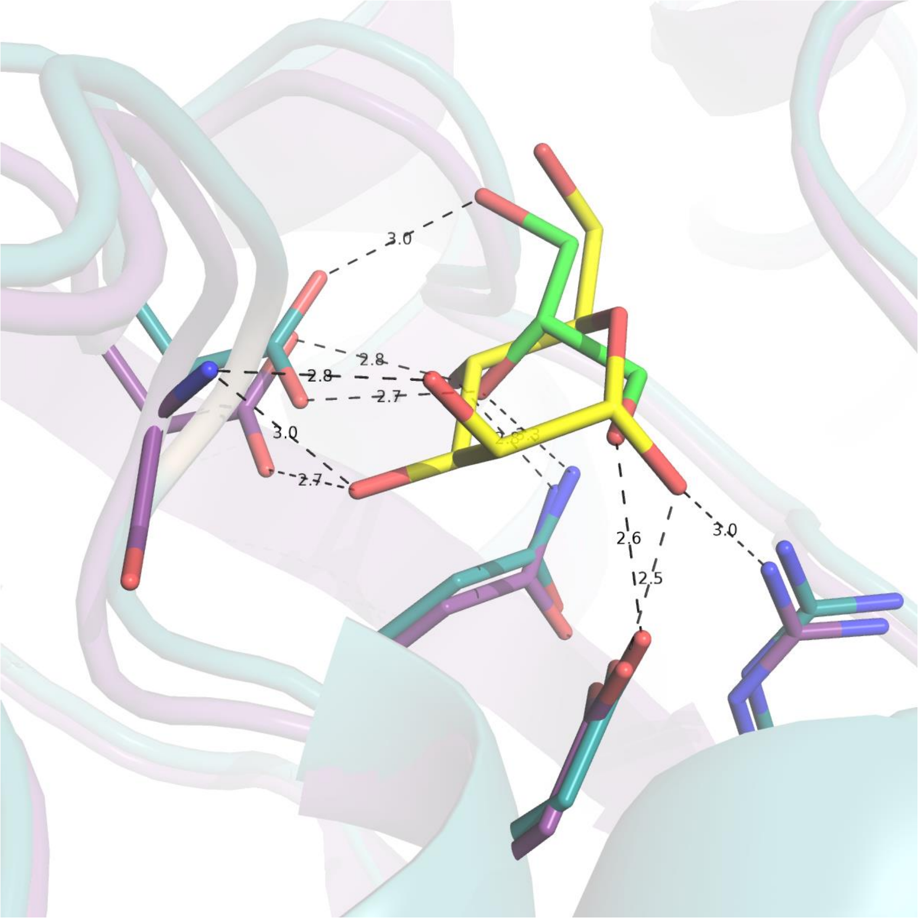

Supplementary Figure 5: Product inhibition studies reveal that WcbL uses an ordered sequential Bi-Bi reaction mechanism (Figure 4). The WcbL reaction was performed in the presence of varying amounts of the product ADP, which clearly inhibited the reaction. **A.** Reactions with differing concentrations of ATP were performed at a constant concentration of 250 μM M7P, and four concentrations of ADP. **B.** Reactions with differing concentrations of M7P were performed at a constant concentration of 1000 μM ATP, and four concentrations of ADP. **C.** The data were fit to three different inhibition models (competitive, non-competitive and uncompetitive). The fit of the best fitting model is indicated in **A** and **B**. The fits were compared using Akaike's Information Criteria, performed in GraphPad version 6.0.2. The level of certainty of the preferred model against each of the other models is indicated. Error bars represent standard error of the mean.

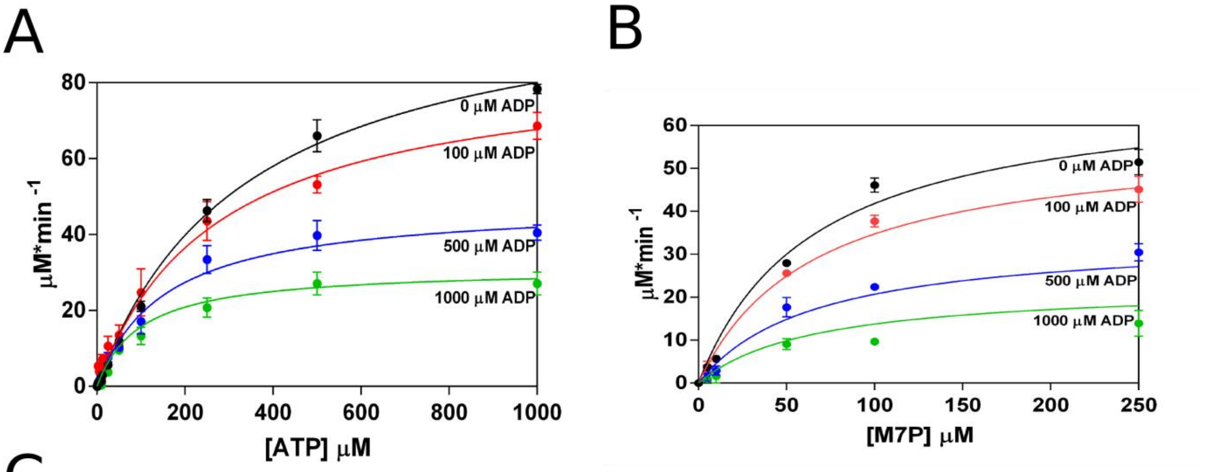

C

Varying [ATP]		Varying [M7P]	
Uncompetitive vs.:		Non-competitive vs.:	
Competitive	99.99%	Competitive	99.99%
Non-competitive	99.99%	Uncompetitive	99.98%

Supplementary Figure 6: Hydrogen bond dilution of WcbL in complex with partner compounds (Figure 5). The hydrogen-bonding network of WcbL was assessed in the absence of ligands (**A**), or the presence of one ATP molecule (**B**), two ATP molecules (**C**), two mannose molecules (**D**), two mannose and one ATP molecules (**E**) and two molecules each of mannose and ATP (**F**). Explicit hydrogens were added to the structure, and the structure optimized, using the online version of YASARA (Krieger, et al., 2009). Hydrogen bonding dilution was performed using PROFLEX (Jacobs, et al., 2001). Briefly, this method identifies hydrogen bonds from the geometry between the four atoms involved in the hydrogen bond. An energy function is then used to rank (i.e. determine the energy of) the hydrogen bonds, depending on the distances and angles between the atoms, and the chemistry of the donor and acceptor. Following this ranking, amino acids that form a network are identified. The weakest hydrogen bonds are then removed, and the networks recalculated. As the hydrogen bonds are diluted (i.e. weaker bonds removed), the networks become progressively smaller, highlighting which networks are most stable. This approach was used to highlight strongly and weakly bonded regions of the protein. The output from the hydrogen bonding dilution is shown, with sections of protein forming durable networks shown in the same color. The three main regions that bind to ATP are indicated by the red, green and blue bars to the left of each image.

In the ligand free state (**A**), both protomers show equivalent rigidity. The rigidity of the protein is increased with the binding of one ATP molecule (**B**: ~0.2 kcal/mol greater stability of the bound protomer) or two mannose molecules (**D**: ~0.1 kcal/mol greater stability compared to the one ATP bound form). This rigidity is lost upon binding of a second ATP (**C**: ~0.3 kcal/mol less stable than the *apo*-structure). A similar effect is observed upon binding one or two ATP molecules to the mannose-bound structure (**E, F**): little difference is seen on binding the second ATP when mannose is bound.

A: Native

Wed Feb 4 13:04:13 2015

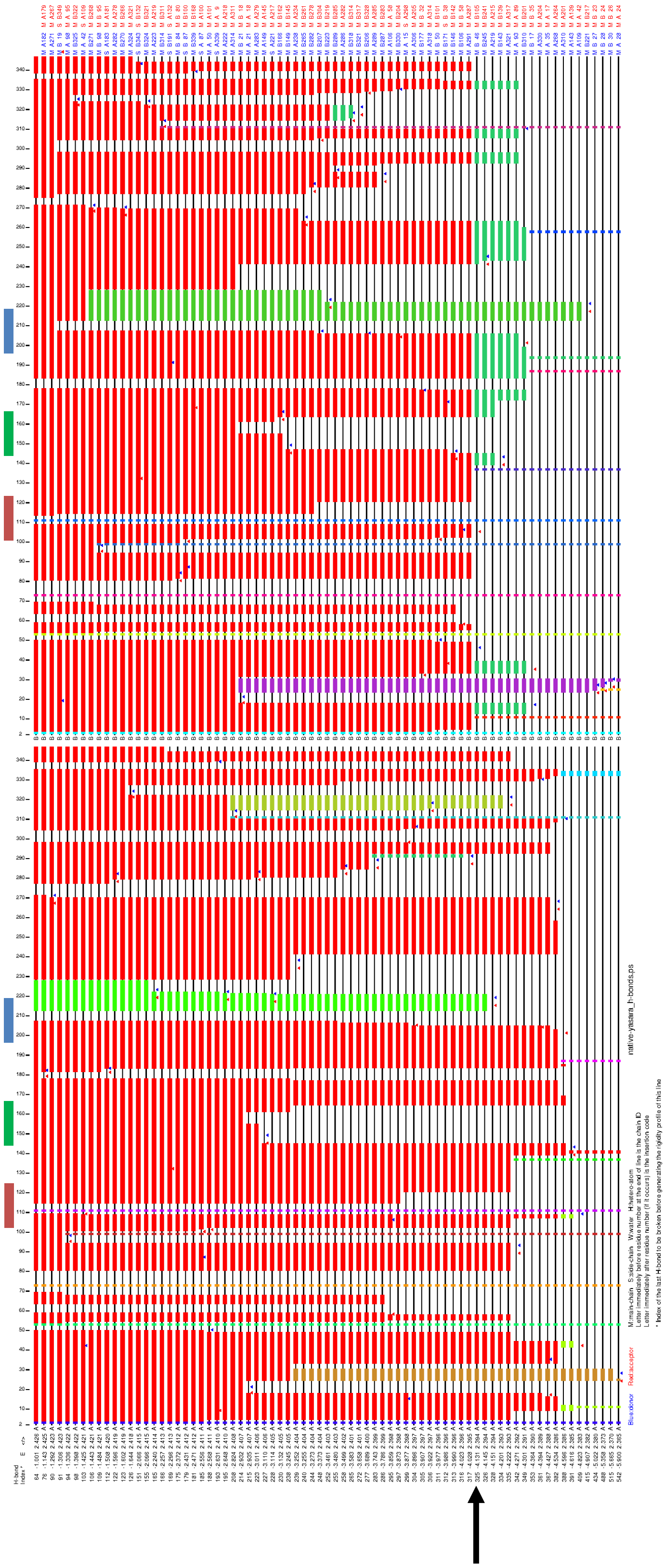

B: 1 ATP (molecule 1)

Wed Feb 4 13:13:33 2015

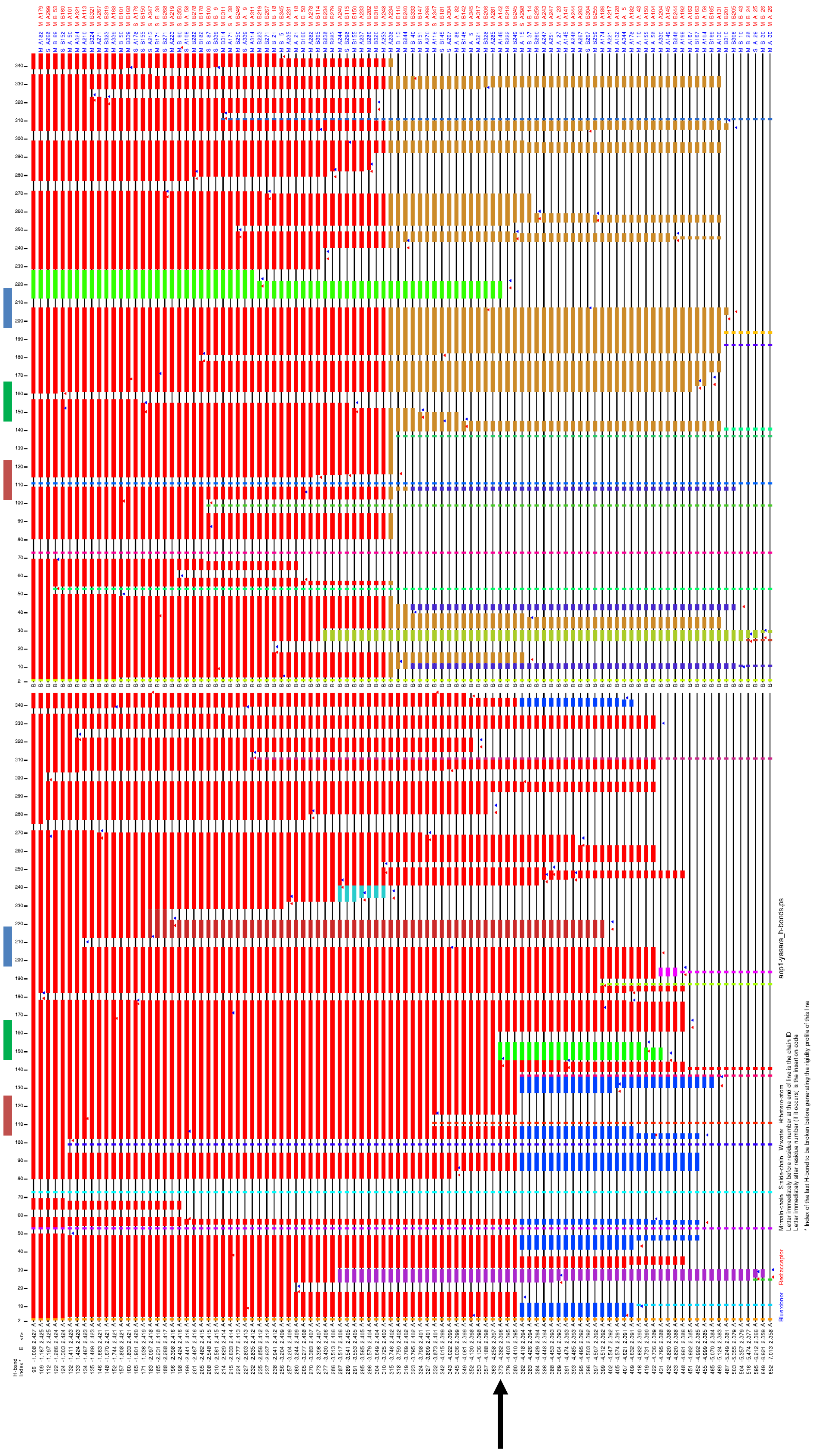

C: 2 ATP

Wed Feb 4 15:42:36 2015

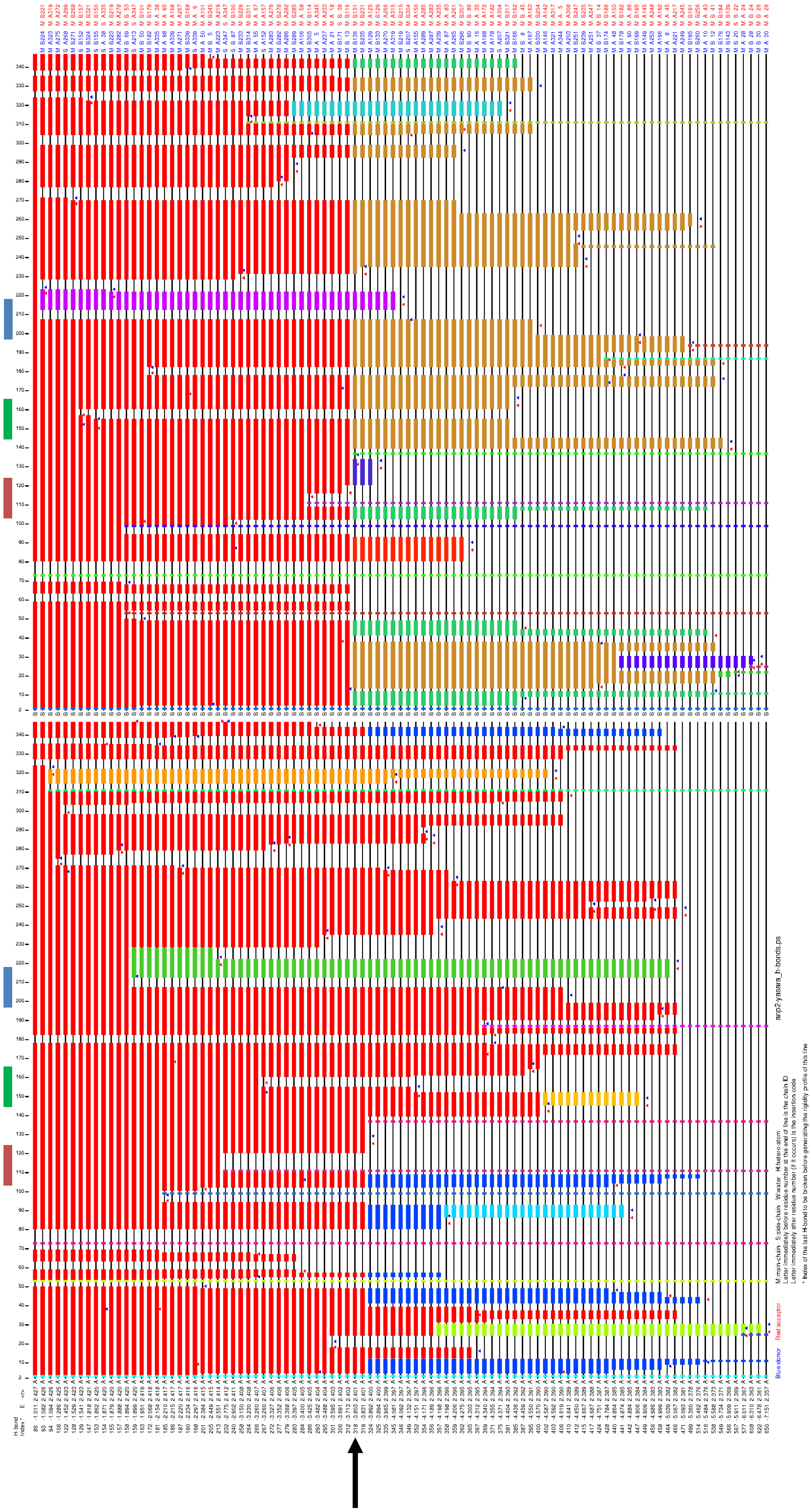

D: 2 mannose

Wed Feb 4 15:59:03 2015

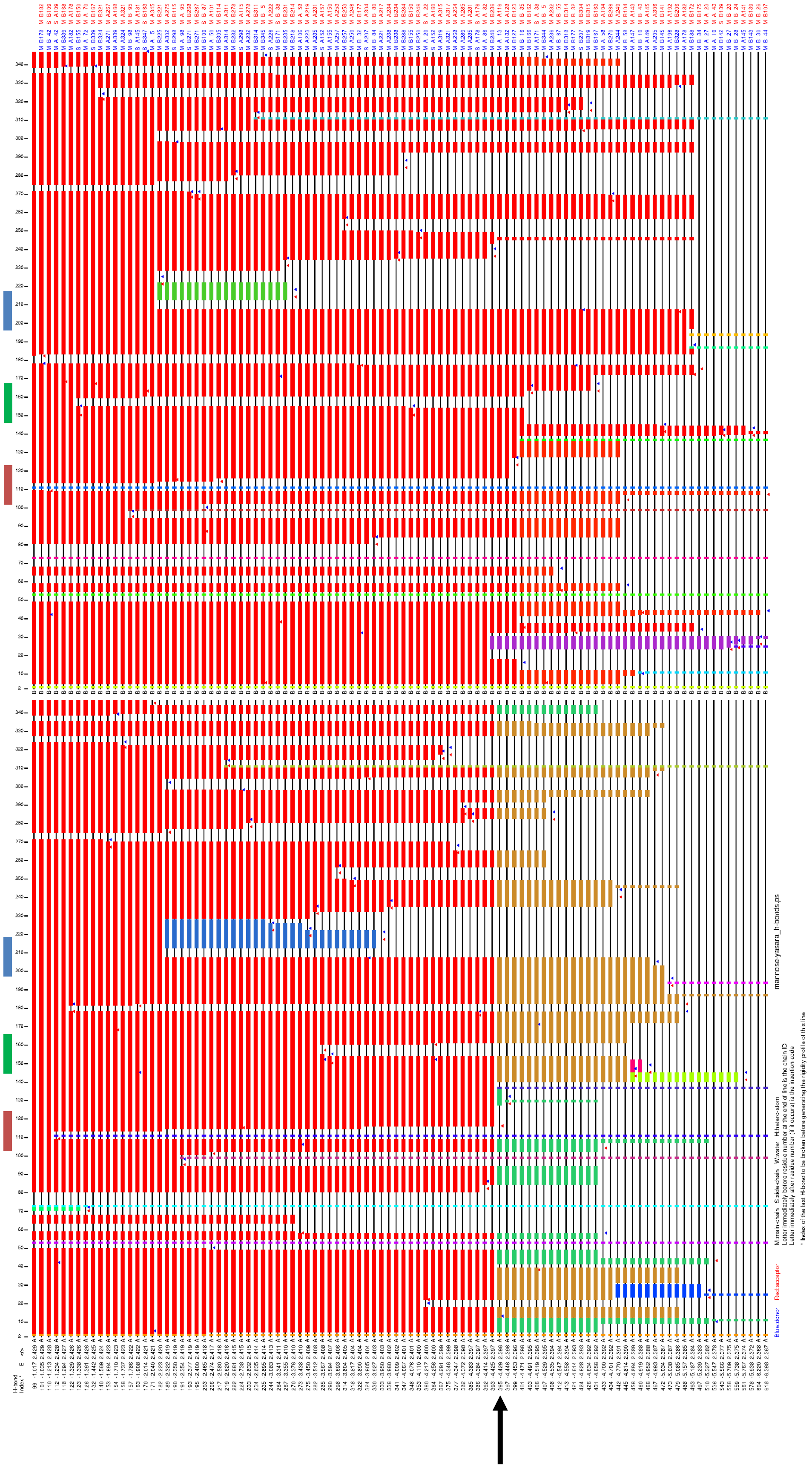

E: 1 ATP (molecule 2) and 2 mannose:

Wed Mar 4 10:30:01 2015

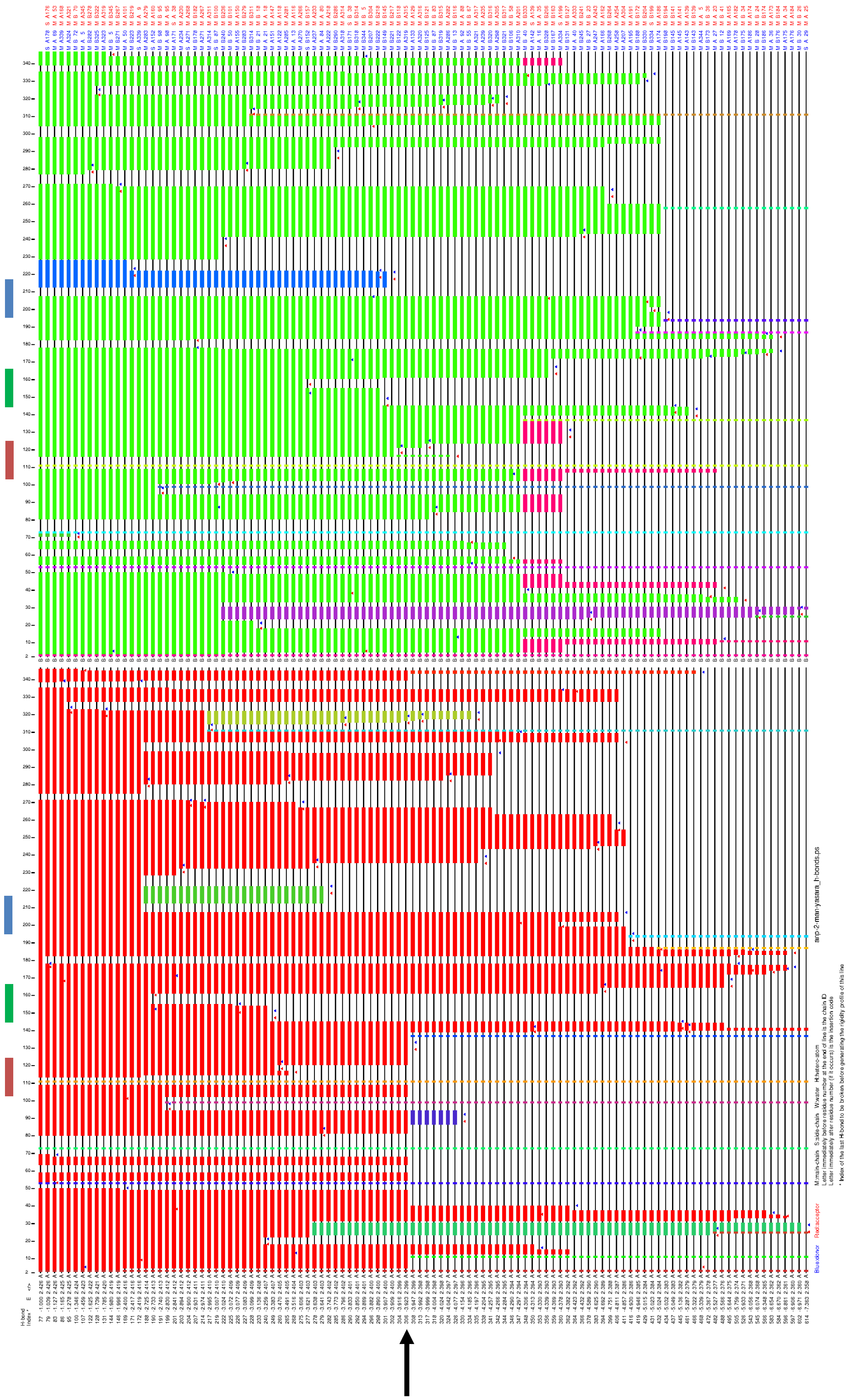

F: 2 ATP and 2 mannose

Wed Feb 4 16:07:42 2015

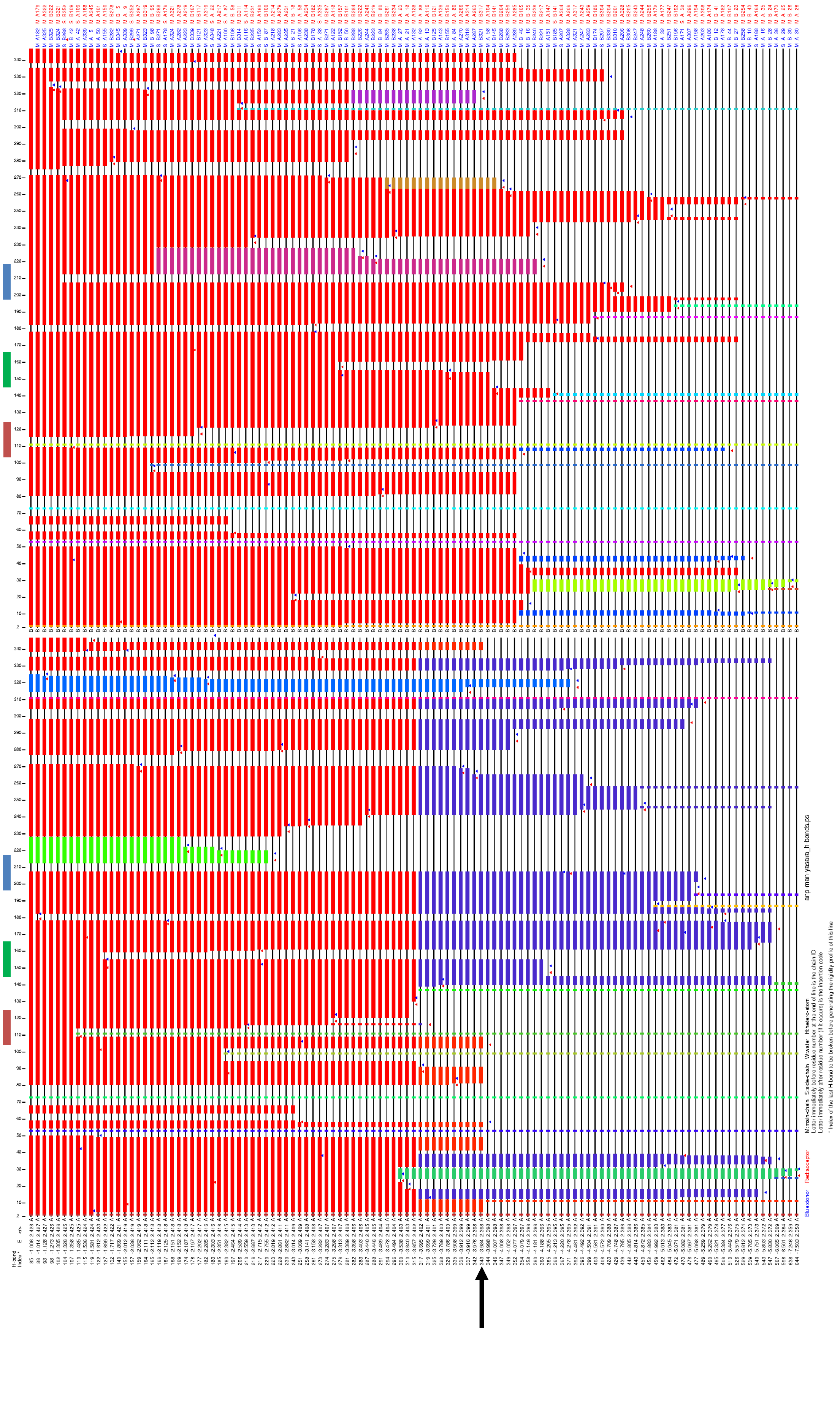

Supplementary references

Chen, V.B., Arendall, W.B., 3rd, Headd, J.J., Keedy, D.A., Immormino, R.M., Kapral, G.J., Murray, L.W., Richardson, J.S., and Richardson, D.C. (2010). MolProbity: all-atom structure validation for macromolecular crystallography. Acta Crystallogr D Biol Crystallogr 66, 12-21.

Jacobs, D.J., Rader, A.J., Kuhn, L.A., and Thorpe, M.F. (2001). Protein flexibility predictions using graph theory. Proteins 44, 150-165.

Krieger, E., Joo, K., Lee, J., Lee, J., Raman, S., Thompson, J., Tyka, M., Baker, D., and Karplus, K. (2009). Improving physical realism, stereochemistry, and side-chain accuracy in homology modeling: Four approaches that performed well in CASP8. Proteins 77, 114-122.

Vaguine, A.A., Richelle, J., and Wodak, S.J. (1999). SFCHECK: a unified set of procedures for evaluating the quality of macromolecular structure-factor data and their agreement with the atomic model. Acta Crystallogr D Biol Crystallogr 55, 191-205.

Yang, J., Yan, R., Roy, A., Xu, D., Poisson, J., and Zhang, Y. (2015). The I-TASSER Suite: protein structure and function prediction. Nat Methods 12, 7-8.
